# Context‐dependent TREM2 signalling in myeloid cells: Molecular mechanisms and immunotherapeutic divergence in neurodegeneration and cancer

**DOI:** 10.1002/ctm2.70863

**Published:** 2026-10-13

**Authors:** Yaqing Lv, Mingpeng Shi, Yongfei Zhao, Yi Yu, Jiawei Lv, Hai Zhao

**Affiliations:** ^1^ Department of Operation Room the Affiliated Hospital of Qingdao University Qingdao Shandong China; ^2^ Department of Neurosurgery the Affiliated Hospital of Qingdao University Qingdao Shandong China; ^3^ Department of Radiology the Affiliated Hospital of Qingdao University Qingdao Shandong China

**Keywords:** Alzheimer's disease, cancer immunotherapy, glioblastoma, lipid metabolism, microglia, TREM2, tumour‐associated macrophages

## Abstract

**Background:**

Triggering receptor expressed on myeloid cells 2 (TREM2) is a pivotal lipid‐sensing receptor that governs the functional plasticity and metabolic fitness of mononuclear phagocytes, including microglia, osteoclasts, dendritic cells and tissue‐resident macrophages. While originally identified as a genetic risk factor for Alzheimer's disease (AD), TREM2 functions as a context‐dependent immunometabolic checkpoint rather than a uniform phenotypic driver, exerting divergent biological outputs dictated by distinct tissue microenvironments and ligand repertoires.

**Summary of key contents:**

This review provides a comprehensive synthesis of TREM2 immunobiology, detailing how ligand engagement—including cell‐surface phospholipids and apolipoprotein E (ApoE)—activates the DAP12/DAP10–SYK–PI3K–mTOR signalling axis. This coordinated intracellular cascade instructively drives myeloid adaptation towards metabolically competent disease‐associated microglia (DAM) and lipid‐associated macrophage (LAM) states, sustaining bioenergetics and debris clearance under pathological stress. Across neurodegenerative proteinopathies and acute cerebral ischemia, TREM2 signalling is predominantly neuroprotective by promoting aggregate compaction and mitigating secondary excitotoxicity, although chronic dysregulated activation may drive stage‐dependent neuroinflammation. In peripheral inflammatory pathologies such as rheumatoid arthritis and sepsis, TREM2 fine‐tunes macrophage efferocytosis and systemic metabolic thresholds. In oncology, TREM2 displays a complex, microenvironment‐dependent divergence: in central nervous system malignancies such as glioblastoma (GBM), TREM2 exerts controversial, stage‐dependent effects, spanning microglial tumour containment in early stages versus pro‐tumoural myelin recycling and immunosuppression by monocyte‐derived macrophages in post‐treatment recurrence; conversely, in peripheral solid tumours, monocyte‐derived TREM2+ tumour‐associated macrophages (TAMs) predominantly orchestrate an immunosuppressive, nutrient‐deprived niche that impairs cytotoxic T‐cell function. Furthermore, we synthesise emerging paradigms along the bidirectional microbiome–gut–brain–tumour axis, elucidating how commensal and intra‐tumoural microbial cues dynamically calibrate myeloid signalling tone and therapeutic susceptibility.

**Conclusion:**

We address translation bottlenecks—including blood–brain barrier (BBB) shuttles, human trial scarcity and soluble TREM2 (sTREM2) biomarker harmonisation—outlining a biomarker‐guided, context‐tailored precision therapeutic framework for targeting the TREM2 axis.

## Highlights

- TREM2 acts as a context‐dependent immunometabolic rheostat shaping myeloid functional plasticity across neurodegeneration and cancer.
- Distinct cellular ontogeny, tissue microenvironment and disease stage determine dual, opposing TREM2 outputs in glioblastoma versus peripheral solid tumors.
- Bidirectional microbiome‐gut‐brain‐tumor axis calibrates TREM2‐driven myeloid signaling and modulates responsiveness to TREM2‐targeted immunotherapies.
- TREM2‐based therapeutics face major translational bottlenecks including BBB delivery obstacles, off‐target risks, and lack of standardized sTREM2 biomarker assays.
- Biomarker‐guided, context‐tailored precision strategies are required rather than universal TREM2 agonism or blockade for clinical translation.

## Graphical abstract

- Divergent triggering receptor expressed on myeloid cells 2 (TREM2) signalling pathways instruct myeloid cell adaptation towards neuroprotective disease‐associated microglia (DAM)/lipid‐associated macrophage (LAM) or immunosuppressive tumour‐associated macrophage (TAM) phenotypes.
- The functional outcome is dictated by distinct microenvironments, ligand availability and systemic microbial cues along the gut–brain–tumour axis.
- Precision therapeutic optimisation requires overcoming translation barriers like blood–brain barrier shuttling and standardised sTREM2 biomarker harmonisation.

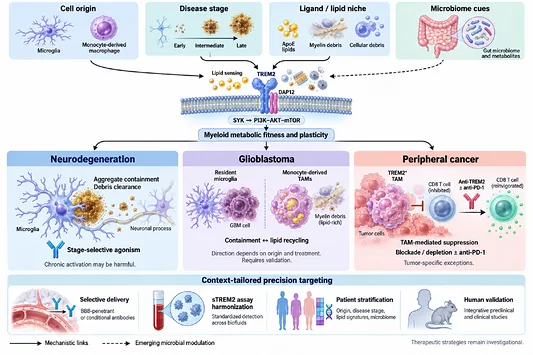

## 1 INTRODUCTION

Triggering receptor expressed on myeloid cells 2 (TREM2) is an immunoglobulin superfamily surface receptor predominantly restricted to mononuclear phagocytes, including tissue‐resident macrophages, osteoclasts, dendritic cells (DCs) and central nervous system (CNS) microglia.^1^ While initially identified as a strong genetic risk factor for late‐onset Alzheimer's disease (AD), subsequent genomic, single‐cell transcriptomic and functional studies have propelled TREM2 to the forefront of immunobiology, revealing its fundamental role as an instructive immunometabolic and lipid‐sensing checkpoint across diverse human pathologies.^2^, ^3^, ^4^

Despite rapid clinical translation, the mechanistic landscape of TREM2 presents a profound biological conundrum: how does the engagement of an identical receptor complex yield diametrically opposed immunophenotypic outcomes across different pathological ecosystems? In chronic neurodegenerative proteinopathies, TREM2‐dependent lipid scavenging and metabolic fitness are generally required to license barrier containment and aggregate clearance; yet, under persistent pathological stress, chronic signalling can paradoxically accelerate neurotoxic inflammation.^5^, ^6^, ^7^ Conversely, across peripheral and intracranial malignancies, the functional identity of TREM2‐expressing tumour‐associated macrophages (TAMs) spans a polarising spectrum—from microglial tumour containment to treatment‐induced immunosuppression, pro‐tumoural lipid recycling and resistance to immune checkpoint blockade (ICB).^8^, ^9^, ^10^, ^11^ Furthermore, emerging evidence demonstrates that TREM2 biology is not confined within isolated organ borders, but is dynamically calibrated by external systemic drivers along the bidirectional gut–brain–tumour axis.^12^

Resolving these contradictory phenotypes requires moving decisively beyond oversimplified binary dogmas.^13^, ^14^, ^15^ A unified mechanistic framework must instead dissect how cell ontogeny, spatial lipid architecture, receptor‐shedding kinetics and microbiome‐derived cues instruct myeloid functional states.^1^, ^16^, ^17^, ^18^, ^19^ In this review, we synthesise recent structural, mechanistic and translational advances in TREM2 immunobiology. We specifically address the contentious phenotypic debates across neurodegenerative and oncologic niches, delineate the regulatory logic of the gut–brain–tumour axis, and critically evaluate the formidable pharmacokinetic, analytical and immunological barriers that must be surmounted to realise precision TREM2‐directed therapeutics.

## 2 SEARCH STRATEGY AND SELECTION CRITERIA

Literature retrieval was performed across PubMed, Web of Science, Embase and Scopus databases for peer‐reviewed articles published up to 2026. The search utilised combinations of MeSH terms and text words including: ‘TREM2’, ‘Triggering Receptor Expressed on Myeloid Cells 2’, ‘microglia’, ‘tumour‐associated macrophages’, ‘disease‐associated microglia’, ‘lipid‐associated macrophages’, ‘Alzheimer's disease’, ‘glioblastoma’, ‘cancer immunotherapy’, ‘gut–brain axis’ and ‘soluble TREM2’. Priority was given to high‐impact original research papers and mechanistic reviews published within the last 5 years, supplemented by foundational structural, genetic and developmental reports. Only English‐language, peer‐reviewed publications were incorporated.

## 3 BIOLOGY OF TREM2

TREM2 is a membrane‐spanning receptor that functions as a key regulator of innate immune signalling. First characterised in 2000, it was identified as a cell‐surface molecule predominantly expressed by myeloid‐lineage cells, including macrophages, DCs and microglia^16^, ^20^, ^21^ (Figure 1). TREM2 is a member of the immunoglobulin superfamily and was originally characterised for its role in regulating microglial activation and neuroinflammatory responses. Over the past two decades, its functional landscape has expanded considerably, with accumulating evidence implicating TREM2 in a wide range of pathological conditions extending beyond the CNS, including AD, malignancies, autoimmune disorders and infectious diseases. ^17^, ^18^

**FIGURE 1:**
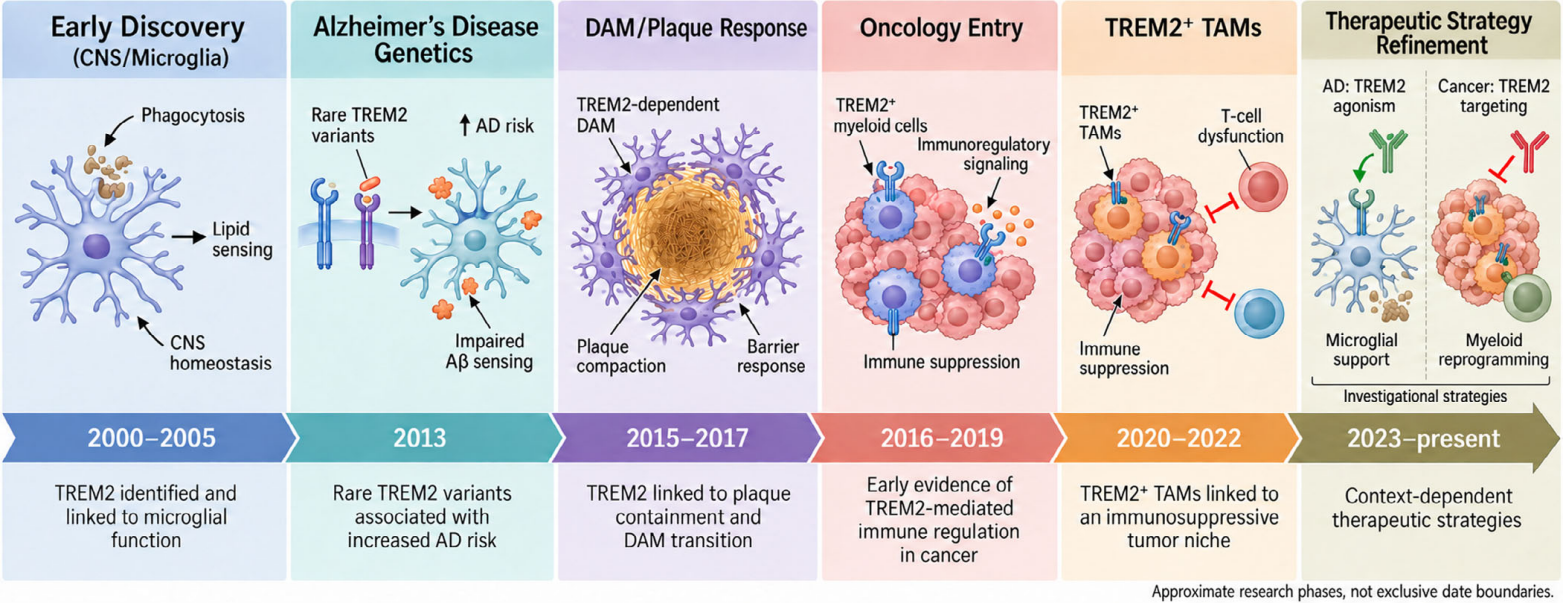
Evolution of TREM2 research from microglial biology to context‐dependent therapeutic strategies. The timeline summarises six approximate phases in the development of research on TREM2. Early studies (2000–2005) established its involvement in microglial phagocytosis, lipid sensing and central nervous system homeostasis. In 2013, rare TREM2 variants were associated with increased AD risk, highlighting the relevance of altered microglial responses to Aβ. Subsequent work (2015–2017) linked TREM2 signalling to plaque compaction, microglial barrier formation and the transition towards DAM. Early oncology studies (2016–2019) implicated TREM2‐expressing myeloid cells in immunoregulatory signalling within tumours. Research during 2020–2022 further characterised TREM2‐positive TAMs and their association with immunosuppressive tumour niches and T‐cell dysfunction. The final phase (2023–present) illustrates the refinement of context‐dependent therapeutic strategies, including TREM2 agonism to support microglial function in AD and TREM2 targeting to reprogram tumour‐associated myeloid cells in cancer. These approaches remain investigational, with effects dependent on disease context and stage. Dates indicate approximate research phases rather than exclusive chronological boundaries or the first appearance of each concept. Arrowheads indicate the relationships depicted, whereas red blunt‐ended lines denote inhibition. Aβ, amyloid‐β; AD, Alzheimer's disease; DAM, disease‐associated microglia; TAMs, tumour‐associated macrophages; TREM2, triggering receptor expressed on myeloid cells 2.

### 3.1 Structural features of TREM2

TREM2 is composed of three principal structural regions: an extracellular immunoglobulin‐like domain, a single‐pass transmembrane segment and a short intracellular cytoplasmic tail.^5^ The extracellular region of TREM2 harbours a single immunoglobulin‐like V‐type (IgV) domain that mediates ligand recognition, including interactions with lipid‐associated molecules and components of apoptotic cells.^5^ High‐resolution structural studies have revealed that the IgV domain of TREM2 adopts a β‐sandwich fold typical of immunoglobulin superfamily receptors. The ligand‐binding interface is formed by surface‐exposed loops enriched in positively charged residues, generating an electrostatic surface optimised for interaction with anionic phospholipids and lipid‐associated apolipoproteins. This structural feature distinguishes TREM2 from certain other immunoglobulin superfamily receptors (e.g., TREM1) that possess both IgV and IgC domains.^19^ The AD‐associated R47H substitution occurs within the IgV domain and replaces a positively charged arginine with histidine, thereby altering the electrostatic surface of the ligand‐binding interface and weakening ligand affinity. Structural modelling suggests that this substitution destabilises receptor–ligand engagement and impairs clustering‐induced signalling, providing a mechanistic explanation for diminished microglial activation observed in carriers. This structural configuration permits TREM2 to engage a diverse repertoire of ligands, including anionic lipids, apolipoproteins such as apolipoprotein E (ApoE) and ApoJ, and phosphatidylserine exposed on apoptotic cells, as evidenced in microglial models.^1^, ^22^ In TAMs, lipid‐rich necrotic debris can function as endogenous TREM2 agonists, engaging the IgV domain and initiating downstream DAP12–SYK–mediated signalling cascades.^9^, ^23^, ^24^ The conformation of the extracellular domain is essential for effective ligand recognition and binding, underpinning TREM2's capacity to modulate immune signalling.

The transmembrane segment secures TREM2 within the plasma membrane, whereas its relatively short cytoplasmic tail contains the elements required for intracellular signal propagation. Notably, unlike many immune receptors, the TREM2 cytoplasmic region lacks a canonical immunoreceptor tyrosine‐based activation motif (ITAM).^25^ Instead, its signalling is primarily mediated by the adapter protein DAP12, which contains an ITAM that becomes phosphorylated upon TREM2 activation.^26^ This phosphorylation event triggers downstream signalling pathways, particularly the phosphoinositide 3‐kinase/protein kinase B/mammalian target of rapamycin (PI3K–AKT–mTOR) axis, which promotes cell survival, immune modulation and anti‐inflammatory responses^26^ (Figure 2). Whereas membrane‐bound TREM2 transduces signals through DAP12‐dependent intracellular pathways, the soluble form (sTREM2) operates in a paracrine manner to promote microglial survival and augment cytokine release.^27^, ^28^ However, within the tumour microenvironment (TME), the balance between soluble and membrane‐bound TREM2 on macrophages remains insufficiently characterised, although increased sTREM2 levels have been associated with immunosuppressive reprogramming of myeloid cells.^29^

**FIGURE 2:**
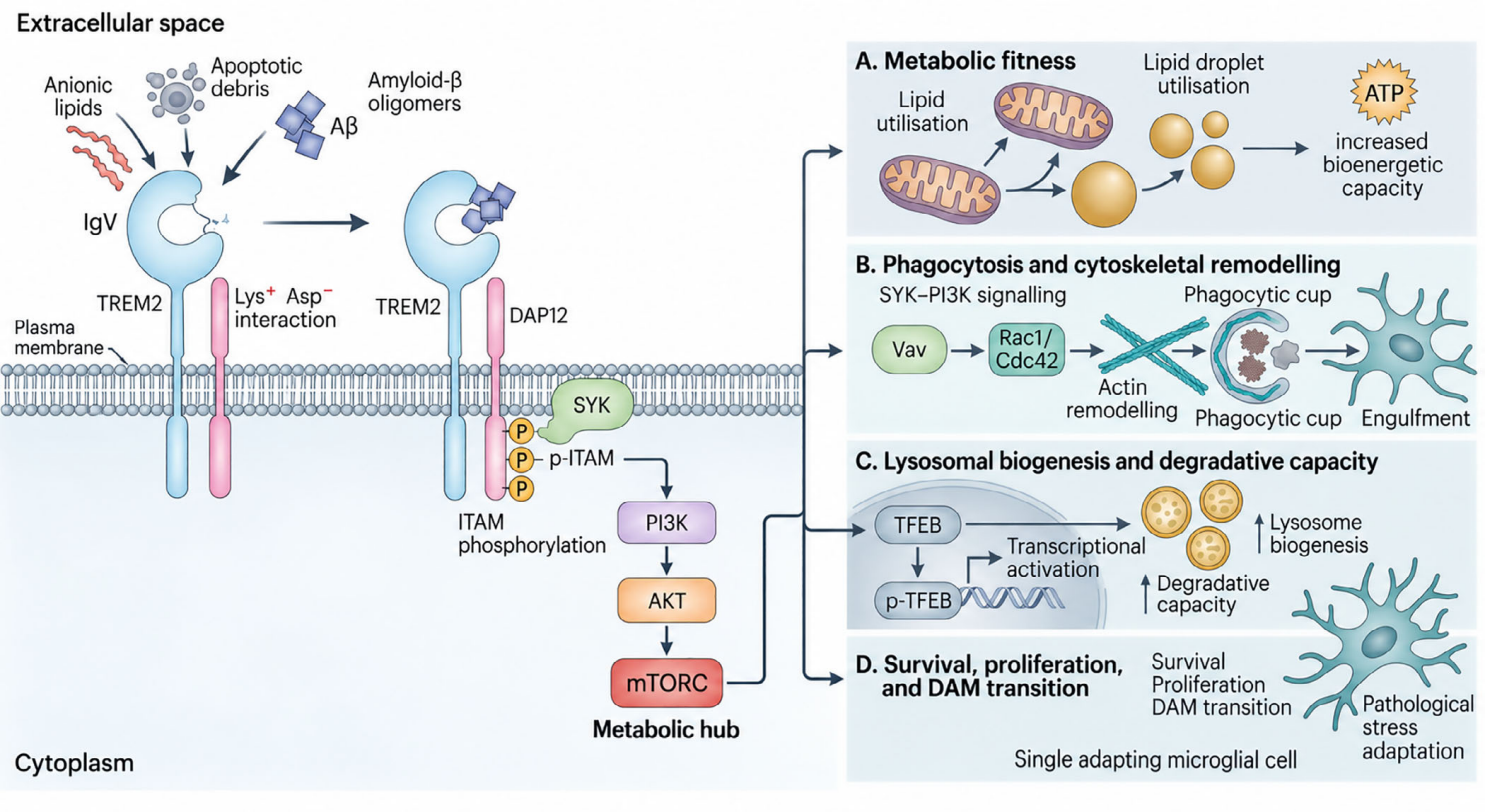
Overview of the TREM2–DAP12 signalling axis and its downstream metabolic and functional programs. This schematic depicts the full signalling cascade of the TREM2 receptor complex, from ligand engagement at the cell surface to downstream effector responses. (A) At the plasma membrane, TREM2 (light blue) associates with the DAP12 adaptor dimer (pink) through electrostatic interactions between positively charged lysine residues within the TREM2 transmembrane region and negatively charged aspartate residues in DAP12. The extracellular IgV domain of TREM2 binds a broad spectrum of ligands, including anionic lipids, apoptotic debris and Aβ oligomers. (B) Ligand binding triggers phosphorylation of ITAMs within the cytoplasmic domains of DAP12, enabling recruitment and activation of SYK (light green). (C) Activated SYK propagates downstream signalling through sequential activation of PI3K (light purple) and AKT (light orange), culminating in stimulation of the mTORC (red). (D) As a central metabolic hub, mTORC coordinates key cellular outputs, including enhanced metabolic activity and bioenergetic capacity, increased phagocytosis, promotion of survival and proliferation, and cytoskeletal remodelling. Specifically, the activation of the SYK–PI3K axis promotes the recruitment of Vav family proteins, which function as guanine nucleotide exchange factors for Rho GTPases. This process triggers actin cytoskeleton reorganisation, providing the mechanical driving force necessary for phagocytic cup formation and the engulfment of amyloid aggregates or apoptotic debris. Simultaneously, mTORC‐ and TFEB‐dependent signalling enhances the biogenesis of lysosomes by regulating the nuclear translocation of TFEB, thereby coupling the metabolic status of the myeloid cell to its degradative capacity. Collectively, this signalling axis underpins the transition of microglia towards a disease‐associated state and sustains their metabolic fitness under pathological stress. Aβ, amyloid‐β; AKT, protein kinase B; Cdc42, cell division control protein 42 homolog; DAP12, DNAX‐activating protein of 12 kDa; IgV, immunoglobulin‐like V‐type; ITAMs, immunoreceptor tyrosine‐based activation motifs; mTORC, mechanistic target of rapamycin complex; PI3K, phosphoinositide 3‐kinase; Rac1, Ras‐related C3 botulinum toxin substrate 1; Rho GTPases, Ras homolog guanosine triphosphatases; SYK, spleen tyrosine kinase; TFEB, transcription factor EB; TREM2, triggering receptor expressed on myeloid cells 2.

### 3.2 Biological functions of TREM2

TREM2 serves as a critical regulator of immune signalling, especially within myeloid‐lineage cells.^26^ Upon ligand binding, TREM2 activates several intracellular signalling cascades that mediate diverse biological functions. Mechanistically, TREM2 signalling via DAP12 promotes SYK phosphorylation and activates the PI3K–AKT–mTOR axis, which enhances cellular metabolic fitness, cytoskeletal rearrangement and phagocytic capacity in myeloid cells.^30^ One of the key roles of TREM2 is in regulating macrophage and microglial activation.^6^, ^31^ In microglia, activation of TREM2 promotes phagocytic capacity, particularly the efficient clearance of apoptotic cells and cellular debris, thereby preserving tissue homeostasis and limiting chronic inflammatory responses.^32^ This function is particularly critical in neurodegenerative diseases (NDDs), where impaired removal of neurotoxic substrates—such as amyloid‐β plaques in AD—drives disease advancement.^32^, ^33^

Beyond microglia and TAMs, TREM2 is functionally expressed across a broader spectrum of myeloid‐lineage cells, governing tissue‐specific homeostasis and remodelling programs.^17^ In bone biology, TREM2 forms an essential signalling complex with DAP12 on osteoclast precursors, where it promotes cell survival, cytoskeletal organisation and multinucleated osteoclast differentiation. Disruption of this physiological axis underlies the severe systemic cystic osteodysplasia observed in Nasu–Hakola disease, highlighting its indispensable role in bone resorption and skeletal remodelling. In peripheral visceral tissues, TREM2 is abundantly expressed by specialised tissue‐resident macrophages, including hepatic Kupffer cells, alveolar macrophages and adipose tissue macrophages (ATMs). Within these metabolic niches, TREM2 functions as a central lipid sensor that buffers tissue lipid overload, clears lipotoxic debris and maintains organ‐level metabolic homeostasis.^34^ In metabolic dysfunction‐associated steatohepatitis and obesity, TREM2+ lipid‐associated macrophages form crown‐like structures to buffer adipocyte lipotoxicity, while scar‐associated macrophages in the liver facilitate the efferocytotic clearance of dying hepatocytes to restrict chronic fibrotic progression.

In secondary lymphoid organs and mucosal barriers, TREM2 expression on specialised DC subsets modulates antigen presentation thresholds, costimulatory molecule expression and maturation kinetics through balanced DAP10 and DAP12 co‐signalling.^7^ Furthermore, under pathological conditions, TREM2 is robustly upregulated on infiltrating myeloid‐derived suppressor cells, where it acts as an instructive metabolic driver that reinforces their suppressive potency against T‐cell‐mediated surveillance.^35^ Beyond its role in phagocytosis, TREM2 has been implicated in directing macrophage functional programming and phenotypic adaptation across a multidimensional activation spectrum.^36^ Rather than conforming to a rigid binary classification of pro‐inflammatory versus anti‐inflammatory activation (historically termed M1/M2), macrophages exhibit remarkable functional plasticity, dynamically tuning their activation programs in response to microenvironmental cues such as tissue lipid accumulation and local cytokine gradients.^37^ Within this continuous phenotypic landscape, TREM2 signalling instructs myeloid cells towards tissue‐resolving and reparative functional states, characterised by enhanced debris clearance, pro‐resolving lipid mediator synthesis and the active dampening of chronic, tissue‐damaging inflammatory signalling.^38^ Through this restorative programming, TREM2 facilitates the timely resolution of acute inflammation and promotes tissue remodelling, a physiological mechanism indispensable not only for CNS homeostasis and neurodegenerative disorders, but also in peripheral inflammatory pathologies such as rheumatoid arthritis (RA) and atherosclerosis^39^, ^40^, ^41^, ^42^ (Figure 3). A fundamental question in myeloid immunobiology is whether TREM2 functions merely as a passive phenotypic marker or acts as an instructive driver of macrophage and microglial polarisation.^43^ Compelling functional and transcriptomic evidence demonstrates that TREM2 plays a definitive instructive role by coupling extracellular lipid sensing to master metabolic and epigenetic reprogramming programs. Rather than simply enabling survival, ligand‐induced TREM2 engagement triggers sustained SYK‐dependent phosphorylation, which activates Mammalian Target of Rapamycin Complex 1 (mTORC1) and promotes the nuclear translocation of TFEB and Peroxisome proliferator‐activated receptor gamma (PPARγ)‐driven gene networks.^44^ This active transcriptional cascade instigates de novo fatty acid oxidation (FAO) and lipid droplet (LD) biogenesis, effectively enforcing the transition of homeostatic myeloid cells into specialised disease‐associated microglia (DAM) or lipid‐associated macrophages (LAM). The instructive nature of TREM2 signalling carries profound immunotherapeutic implications: because TREM2 dynamically guides macrophage functional state transitions rather than imposing irreversible myeloid‐lineage commitment, macrophages retain the intrinsic plasticity to shift between diverse activation programs in response to changing environmental cues.^45^, ^46^ Consequently, therapeutic modulation is not restricted to physical cell elimination or cytotoxic depletion.^47^ Instead, targeting the TREM2 axis enables the functional repolarisation and re‐education of immunosuppressive TAMs into immunostimulatory, antigen‐presenting effectors in solid tumours, or the directed metabolic rejuvenation of exhausted microglia in neurodegenerative lesions, offering a versatile paradigm for precision immunomodulation.^48^

**FIGURE 3:**
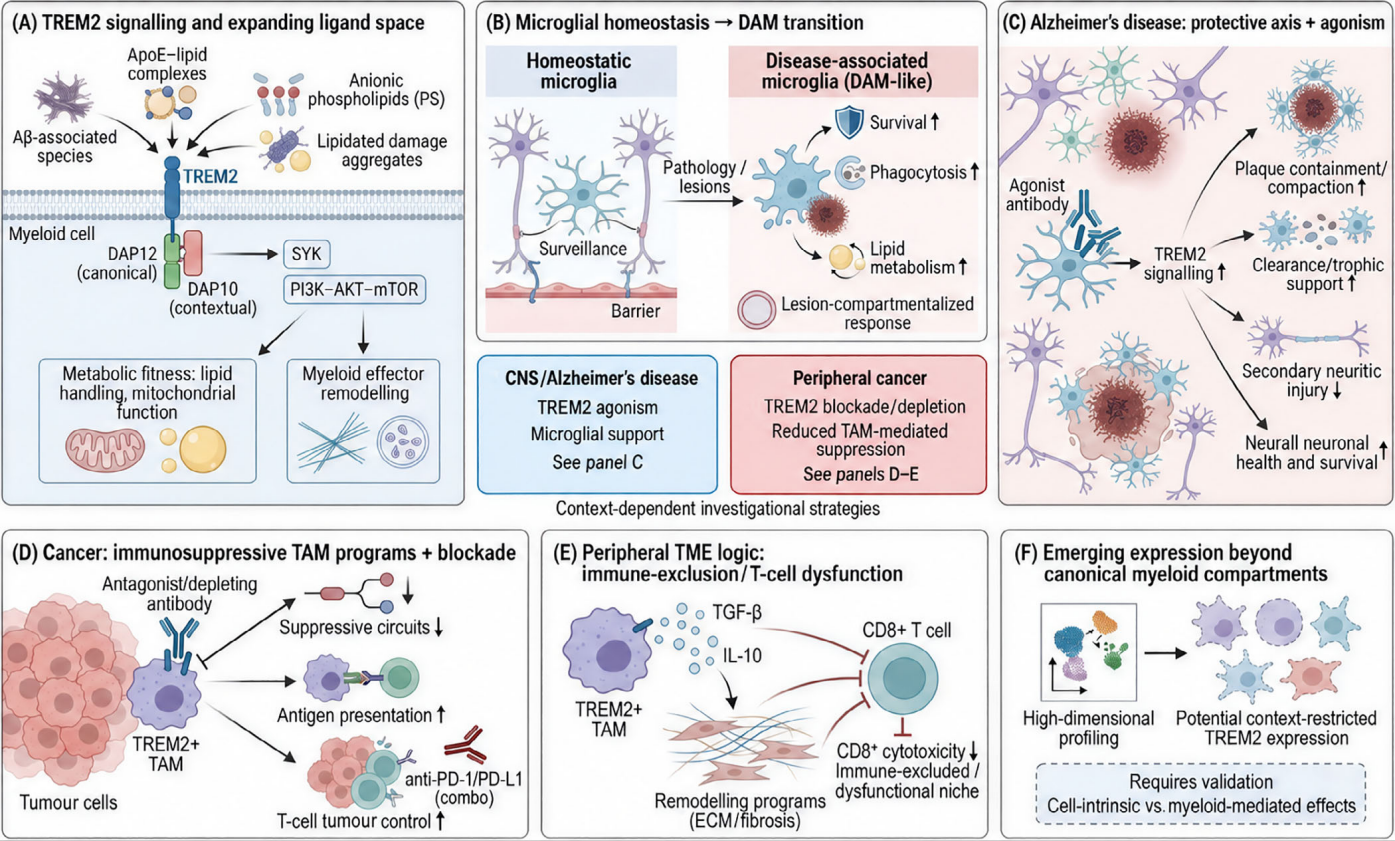
Tissue context shapes TREM2 signalling, myeloid cell functions and therapeutic strategies in neurodegeneration and peripheral cancer. (A) Schematic overview of TREM2 signalling and its expanding ligand landscape, including Aβ‐associated species, ApoE–lipid complexes, anionic phospholipids such as PS, and lipid‐associated damage aggregates. Signalling through the canonical adaptor DAP12, with context‐dependent contributions from DAP10, engages SYK and PI3K–AKT–mTOR pathways to support lipid handling, mitochondrial function and myeloid effector remodelling. (B) Pathological lesions promote the transition from homeostatic microglia towards DAM‐like states, characterised by changes in survival, phagocytosis, lipid metabolism and spatially compartmentalised responses to tissue damage. (C) Proposed protective mechanisms of TREM2 agonism in AD include enhanced plaque containment and compaction, clearance and trophic support, and reduced secondary neuritic injury, collectively supporting neuronal health in relevant experimental settings. (D) In peripheral tumour models, TREM2 blockade or depletion of TREM2‐positive TAMs may attenuate suppressive circuits, enhance antigen presentation and improve T‐cell‐mediated tumour control, including in combination with PD‐1/PD‐L1 blockade. (E) TREM2‐positive TAM‐associated programs may contribute to an immunosuppressive TME through mediators such as TGF‐β and IL‐10 and ECM remodelling, favouring immune exclusion and impaired CD8+ T‐cell cytotoxicity. (F) High‐dimensional profiling suggests potential context‐restricted TREM2 expression beyond canonical myeloid compartments; these observations require validation and distinction between cell‐intrinsic and myeloid‐mediated effects. The central comparison boxes summarise distinct, context‐dependent investigational strategies: microglial support through TREM2 agonism in AD (panel C) and reduction of TAM‐mediated suppression through TREM2 blockade or depletion in peripheral cancer (panels D and E). These strategies do not imply universal benefit or established clinical efficacy. Arrowheads indicate signalling or functional relationships, blunt‐ended lines denote inhibition and upwards and downwards arrows indicate increases and decreases, respectively. Aβ, amyloid‐β; AD, Alzheimer's disease; AKT, protein kinase B; ApoE, apolipoprotein E; CD8, cluster of differentiation 8; DAM, disease‐associated microglia; DAP10, DNAX‐activating protein of 10 kDa; DAP12, DNAX‐activating protein of 12 kDa; ECM, extracellular matrix; IL‐10, interleukin‐10; mTOR, mechanistic target of rapamycin; PD‐1, programmed cell death protein 1; PD‐L1, programmed death‐ligand 1; PI3K, phosphoinositide 3‐kinase; PS, phosphatidylserine; SYK, spleen tyrosine kinase; TAMs, tumour‐associated macrophages; TGF‐β, transforming growth factor‐β; TME, tumour microenvironment; TREM2, triggering receptor expressed on myeloid cells 2.

Although canonical immunobiological paradigms define TREM2 as a receptor strictly restricted to the myeloid lineage, several preliminary reports have described TREM2 expression within lymphoid compartments, including specific subsets of CD4+ T cells, CD8+ T cells and B lymphocytes in severe pulmonary infections and selected tumour contexts.^49^ However, these emerging observations must be interpreted with extreme caution, as the overwhelming majority of large‐scale single‐cell RNA sequencing (scRNA‐seq) and single‐nucleus transcriptomic atlases fail to detect endogenous TREM2 transcripts in pure lymphoid lineages. A rigorous methodological critique suggests that apparent lymphocyte TREM2 positivity in flow cytometry and bulk profiling likely stems from technical artefacts rather than bona fide cell‐intrinsic expression.^50^ Physical cellular doublets—formed by stable immunological synapses and membrane conjugates between tightly bound macrophages or DCs and adjacent T lymphocytes—can easily produce pseudo‐positive signals in high‐throughput cytometry. Additionally, the high phagocytic activity and trogocytosis exhibited by tumour‐infiltrating macrophages can facilitate the transfer of membrane fragments and surface proteins onto neighbouring lymphocytes.^51^ Ambient RNA contamination in low‐input single‐cell droplet systems further contributes to false‐positive transcriptional calls in lymphoid clusters. Until functional promoter reporter assays and cell‐type‐specific conditional knockout models definitively validate de novo lymphocyte protein synthesis, TREM2 should be regarded as a myeloid‐restricted regulator whose apparent presence on lymphocytes predominantly reflects microenvironmental interactions and methodological artefacts.

Recent work has further clarified the mechanistic links between TREM2 signalling and neuroinflammatory regulation.^52^, ^53^

TREM2 activity extends beyond the CNS. In oncology, it has been implicated in shaping the TME through its effects on the phenotype and function of TAMs.^31^, ^54^, ^55^ Analogous to its activity in neurodegenerative settings, TREM2 signalling in TAMs drives an anti‐inflammatory, immunoregulatory phenotype that facilitates immune evasion and supports tumour progression. Paradoxically, in CNS malignancies—most notably glioblastoma (GBM) —the functional role of TREM2 remains highly contentious, with diametrically opposed phenotypes reported across independent laboratories using identical syngeneic models.^3^, ^56^ While early reports suggested that TREM2 agonism or over‐expression enhances immune clearance and cooperates with PD‐1 blockade, rigorous investigations by Sun et al. demonstrated that TREM2 directly orchestrates an immunosuppressive niche in GBM, wherein genetic deficiency or pharmacologic blockade of TREM2 triggers robust myeloid anti‐tumour immunity and significantly extends animal survival.^3^, ^56^

The structural organisation of TREM2 closely aligns with its functional roles. Its extracellular immunoglobulin‐like domain is critical for recognising and engaging lipid‐associated ligands, a prerequisite for mediating its immunomodulatory activity.^57^ Maintaining the structural integrity of the extracellular domain is essential for effective ligand recognition, and mutations within this region can compromise TREM2 function, as observed in AD. Downstream, signal transduction through the DAP12 adaptor converts receptor engagement into coordinated immune responses, including macrophage polarisation and enhanced phagocytic activity.^30^ This tight coupling between structure and function is especially apparent in TREM2's immunoregulatory capacity, whereby its molecular architecture enables environmental signal detection and downstream modulation of immune cell behaviour.

## 4 TREM2 IN AD AND OTHER NDDS

Genetic studies have identified several rare missense mutations in the TREM2 gene that significantly increase susceptibility to late‐onset AD and other NDDs. Among these, the R47H variant is the most strongly associated with AD risk, conferring a two to fourfold increase in susceptibility.^58^, ^59^ This variant impairs ligand recognition and decreases TREM2 surface availability, resulting in defective microglial activation and reduced phagocytic capacity. Individuals carrying the R47H mutation display attenuated responses to Aβ assemblies, accompanied by greater plaque deposition, heightened neuroinflammation and increased synaptic degeneration relative to non‐carriers. Additional variants, including R62H and T66M, similarly destabilise receptor structure and disrupt DAP12–SYK‐dependent signalling, further supporting the view that TREM2 loss‐of‐function mutations potentiate neurodegenerative progression. When encountering Aβ plaques in AD, TREM2‐expressing microglia cluster tightly around amyloid deposits to facilitate plaque compaction and limit the diffusion of toxic Aβ oligomers.^60^, ^61^ This microglial barrier function is markedly reduced in TREM2‐deficient or R47H mutant models, resulting in diffuse, less compacted plaques and heightened neuritic dystrophy. Functionally, high‐resolution single‐cell transcriptomic profiling has established that the transition of homeostatic microglia into the DAM phenotype is not an all‐or‐none event, but rather proceeds through a sequential, two‐step developmental trajectory governed by distinct molecular checkpoints.^62^, ^63^ The initial step, termed Stage 1, is TREM2‐independent. Upon encountering early pathological stressors, homeostatic microglia downregulate canonical homeostatic signature genes while initiating the baseline induction of priming factors including Tyrobp, ApoE and B2m.^63^ However, progression to Stage 2 is strictly TREM2‐dependent. Ligation of the TREM2–DAP12 receptor complex is obligatory to license this secondary transcriptional cascade, which drives the robust upregulation of lipid‐handling enzymes, phagocytic machinery and lysosomal biogenesis programs.^64^, ^65^ In murine models deficient in TREM2 or carrying the human loss‐of‐function R47H variant, microglia become transcriptionally arrested at the intermediate Stage 1 checkpoint.^66^ As a result, these stalled microglia fail to acquire the bioenergetic fitness and metabolic competence required to physically surround, compact and seal neurotoxic amyloid deposits.^67^ A central function of TREM2 in AD lies in its capacity to modulate neuroinflammatory responses.^68^ During neurodegenerative processes, activated microglia frequently secrete pro‐inflammatory cytokines, thereby amplifying the inflammatory milieu characteristic of AD.^69^ Experimental models of AD demonstrate that loss of functional TREM2 leads to exaggerated neuroinflammatory responses and defective resolution of inflammation.^18^ Conversely, enhanced TREM2 expression has been linked to heightened microglial competence, attenuation of neuroinflammatory signalling and more effective clearance of amyloid‐β deposits.^70^ In addition to the membrane‐associated receptor, sTREM2 has gained attention as a biomarker of microglial activation. Increased levels of sTREM2 in cerebrospinal fluid (CSF) are observed during early stages of AD and have been associated with a slower rate of cognitive decline, consistent with a putative compensatory neuroprotective response.^71^, ^72^

Importantly, although TREM2 signalling can attenuate acute neuroinflammatory responses, its precise contribution to the dynamic equilibrium between neuroprotection and neurodegeneration remains incompletely defined. Emerging evidence suggests that chronic or dysregulated TREM2 activation may perpetuate inflammatory signalling, potentially accelerating neurodegenerative progression (Figure 4). Given that AD‐associated variants predominantly confer loss‐of‐function effects, therapeutic strategies primarily focus on TREM2 agonism—utilising agonistic antibodies or ligand‐mimetics—to restore microglial competence and enhance Aβ clearance. Preclinical evaluations demonstrate that TREM2 agonism reinforces plaque compaction, attenuates neuritic dystrophy and limits amyloid‐induced tau seeding and spreading. Early‐phase clinical testing of humanised agonistic antibodies like AL002 has successfully confirmed target engagement and pharmacodynamic shifts in CSF sTREM2, representing a critical translational milestone.^58^ The functional role of TREM2 in tau‐driven neurodegeneration represents one of the most prominent controversies in neuro‐immunology, characterised by starkly contradictory preclinical observations across different mouse models.^58^ In pure tauopathy strains such as the PS19 (tau‐P301S) model, complete genetic ablation of TREM2 has yielded paradoxical outcomes: earlier studies suggested that TREM2 deficiency attenuates tau‐mediated neurodegeneration and brain atrophy, whereas subsequent investigations demonstrated that TREM2 loss exacerbates hyperphosphorylated tau accumulation and cortical synapse loss. These phenotypic discrepancies are critically governed by three interrelated biological determinants: disease staging, baseline microglial metabolic status and TREM2 expression dosage. During the nascent stages of tau seeding and amyloid‐induced pathology, intact TREM2 expression provides vital metabolic support that enables microglia to encapsulate amyloid seeds, limiting secondary amyloid‐induced tau spreading across synaptic circuits.^73^ However, in advanced disease stages or pure tau models lacking amyloid plaques, such as the P301L model, the absence of amyloid‐β eliminates the protective clustering trigger. Under these conditions, sustained TREM2 hyperactivation or forced transgenic over‐expression can drive microglia into a state of metabolic exhaustion and chronic neuroinflammation, accelerating toxic tau propagation along functional neural networks.^74^ Furthermore, the gene dosage of TREM2 imparts a non‐linear effect, wherein severe loss‐of‐function variants like R47H impair protective clearance, while indiscriminate hyperactivation precipitates neurotoxic collateral damage, demonstrating that TREM2 signalling in tauopathy operates along a strictly stage‐dependent and dosage‐sensitive continuum.^75^

**FIGURE 4:**
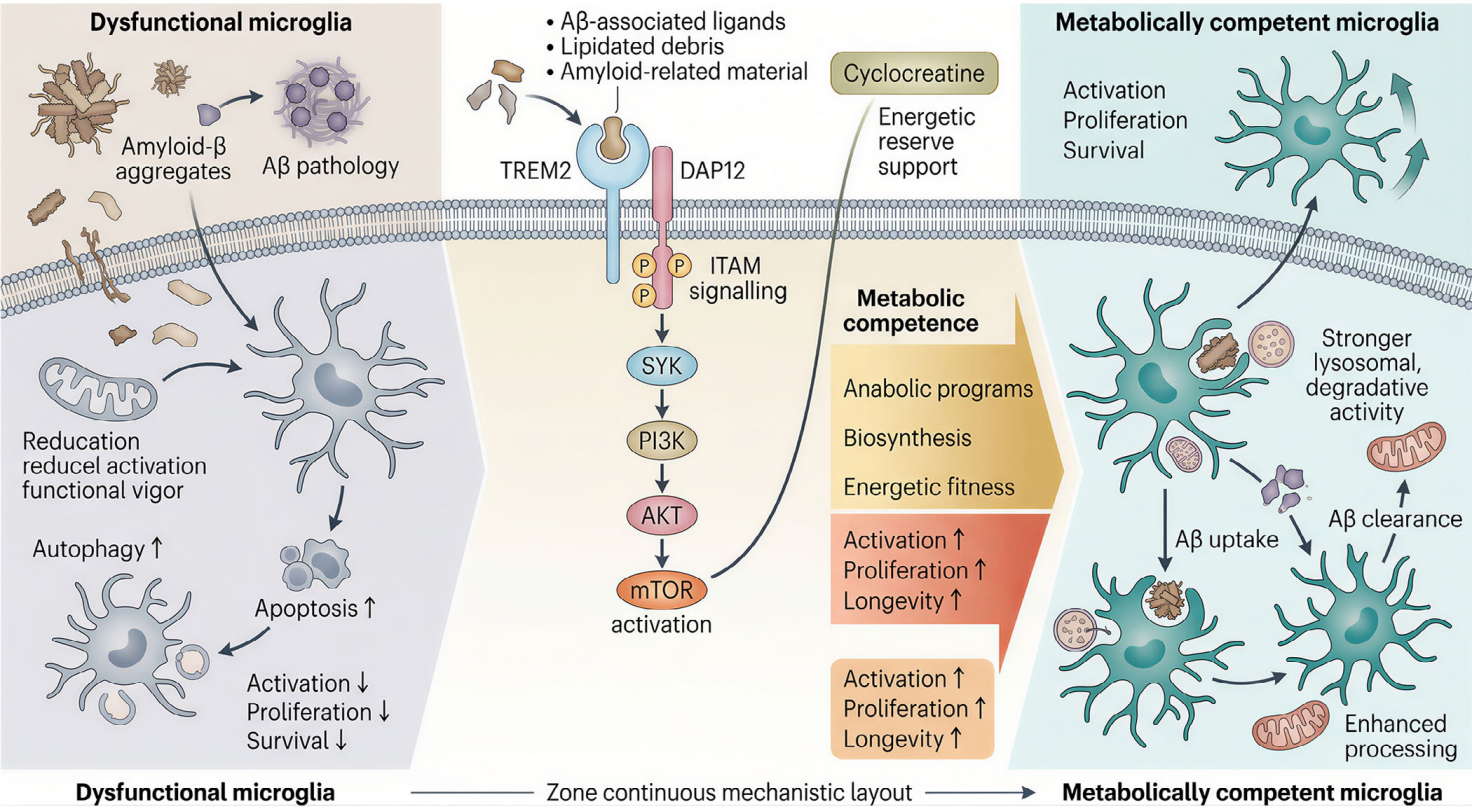
TREM2–DAP12 signalling reinforces microglial metabolic competence and Aβ processing through mTOR activation. This model illustrates how engagement of the TREM2–DAP12 axis supports the establishment of a metabolically ‘competent’ microglial phenotype in the presence of Aβ pathology. On the left, dysfunctional microglia display increased markers of autophagy and apoptosis alongside diminished activation, proliferative capacity and survival. Upon ligand binding, TREM2 associates with DAP12 and initiates ITAM‐dependent signalling, leading to activation of downstream effectors including mTOR. This signalling cascade drives anabolic and biosynthetic programs that enhance microglial activation, proliferation and longevity. The central transition towards a metabolically robust state is depicted, and may be further reinforced by ITAM‐supporting signals—such as cyclocreatine—which augment cellular energetic reserves and improve the capacity of microglia to respond to and clear Aβ aggregates. Aβ, amyloid‐β; DAP12, DNAX‐activating protein of 12 kDa; ITAM, immunoreceptor tyrosine‐based activation motif; mTOR, mechanistic target of rapamycin; TREM2, triggering receptor expressed on myeloid cells 2.

Beyond the pathological context of AD, TREM2‐mediated neuroprotection extends broadly to other neurodegenerative proteinopathies, particularly PD, in which microglial dysfunction critically drives α‐synuclein‐related neurotoxicity and progressive nigrostriatal degeneration.^76^ High‐dimensional single‐nucleus RNA sequencing of human substantia nigra tissue has confirmed that TREM2 expression is selectively remodelled within DAM clusters surrounding vulnerable dopaminergic neurons, directly shaping their ability to clear extracellular α‐synuclein oligomeric fibrils. Large‐scale multi‐centre longitudinal clinical cohorts further validate the translational potential of sTREM2: dynamically altered CSF sTREM2 levels in early‐stage PD patients closely correlate with neuroinflammatory status, clinical motor severity, the rate of motor function decline and reduced putaminal dopamine transporter binding. Consistent with clinical observations, preclinical mechanistic models verify that functional TREM2 deficiency substantially impairs microglial phagocytic clearance of toxic α‐synuclein aggregates, exacerbates dopaminergic neuronal loss and amplifies chronic neuroinflammation. Mechanistically, TREM2 dysfunction further disrupts microglial lipid metabolic homeostasis essential for membrane integrity and debris clearance, thereby destabilising midbrain microenvironmental equilibrium.^77^ Collectively, these findings establish TREM2‐dependent debris clearance and immune‐metabolic regulation as a universal homeostatic mechanism across neurodegenerative proteinopathies, strongly supporting the expansion of TREM2‐targeted therapeutic strategies from classical amyloidopathies to α‐synuclein‐driven Parkinsonian neurodegeneration.

Extending this functional paradigm from subcortical circuits to cortical neurodegenerative syndromes, compromised TREM2 signalling occupies a central position in the pathogenesis of frontotemporal dementia (FTD).^78^ Human genetic sequencing has firmly established that the rare loss‐of‐function TREM2 R47H missense variant confers elevated susceptibility to behavioural variant FTD and broader frontotemporal lobar degeneration (FTLD). In large clinical cohorts, CSF sTREM2 concentrations are dynamically altered in FTD patients carrying pathogenic GRN or C9orf72 mutations, serving as a fluid biomarker reflecting the magnitude of cortical microglial activation.^79^ Mechanistic evidence derived from post mortem spatial transcriptomics (STs) and transgenic animal models confirms that intact TREM2 signalling is indispensable for microglial phagocytic clearance of pathological TDP‐43 aggregates and hyperphosphorylated tau inclusions. TREM2 insufficiency blunts microglial responsiveness to cortical tissue stress and prevents efficient engulfment of toxic protein deposits; because TREM2 fundamentally couples lipid sensing to microglial survival and effector function, its dysfunction drives pronounced regional lipid metabolic disturbances within vulnerable frontal and temporal lobes, triggering persistent lipid accumulation, sustaining unresolved neuroinflammation and accelerating progressive neuronal atrophy.^80^ Notably, TDP‐43 proteinopathy represents a shared molecular hallmark linking FTLD and motor neuron disease, thereby creating a mechanistic molecular bridge between FTD and amyotrophic lateral sclerosis (ALS).

In ischaemic stroke, clinical cohort investigations demonstrate that serum and CSF concentrations of sTREM2 rise sharply following acute arterial occlusion, showing a direct quantitative correlation with infarct volume and serving as a predictive biomarker for 90‐day neurological recovery.^81^ Mechanistically, single‐cell transcriptomic profiling of murine ischaemic brains reveals that TREM2 activation on resident microglia drives an IGF1‐dependent metabolic switch towards enhanced glycolysis and oxidative phosphorylation, promoting efferocytosis of glutamate‐damaged necrotic neurons and accelerating post‐ischaemic tissue repair.^82^

Positioned along this shared clinicopathological spectrum spanning FTLD and motor neuron disease, TREM2 acts as a critical determinant of microglial dynamics, neuroinflammation and motor neuron survival in ALS.^83^ High‐resolution scRNA‐seq profiling of human post mortem spinal cords identifies a distinct TREM2‐dependent DAM subset programmed to clear pathological TDP‐43 inclusions, degenerating motor neurons and toxic myelin debris. Longitudinal clinical cohort studies validate the prognostic value of soluble TREM2: elevated CSF sTREM2 concentrations detected during early ALS independently predict slower motor functional deterioration and prolonged patient survival, establishing sTREM2 as a robust fluid biomarker. Mechanistic data from preclinical models further demonstrate that intact TREM2 signalling sustains microglial metabolic fitness, restricts secondary neurotoxicity along corticospinal motor tracts and governs microglia–neuron crosstalk linked to synaptic pruning. By modulating excessive synaptic elimination, TREM2 activity attenuates pathological neuronal hyperexcitability, revealing a compelling therapeutic window for stage‐selective TREM2 activation aimed at preserving vulnerable spinal and cortical motor circuits.^84^

Beyond the nervous system, TREM2 has emerged as a key regulator in peripheral inflammatory diseases. In RA, scRNA‐seq analysis of human synovial tissue biopsies has identified a unique TREM2+ tissue‐resident macrophage population localised to the synovial lining layer.^85^ These TREM2+ macrophages produce resolving lipid mediators, restrain osteoclastogenesis and correlate with sustained clinical disease remission. Conversely, in sepsis and severe bacterial endotoxemia, human clinical studies demonstrate that elevated plasma sTREM2 reflects systemic monocyte exhaustion.^86^ Preclinical models demonstrate that TREM2 engagement modulates the SH2 domain‐containing protein tyrosine phosphatase 1/Bruton's tyrosine kinase (SHP1/BTK) signalling cascade, fine‐tuning FAO and determining the clinical boundary between protective bacterial clearance and lethal cytokine storm.^12^

## 5 TREM2 AT THE MICROBIOME INTERFACE: BRIDGING THE BIDIRECTIONAL GUT–BRAIN–TUMOUR AXIS

While TREM2 has traditionally been characterised as a cell‐intrinsic lipid receptor, emerging paradigms reveal a far broader systemic dimension: its dynamic and multi‐layered interplay with the host microbiome.^44^ Rather than operating strictly within localised tissue compartments, TREM2 functions as a central molecular bridge within a novel, bidirectional Gut–Brain–Tumour Axis. This interplay operates across two complementary dimensions: systemic immune‐metabolic tuning by the gut microbiota and localised microenvironmental reprogramming by the intra‐tumoural microbiome. Although this cross‐talk represents a cutting‐edge and rapidly expanding frontier at the intersection of microbiology, neurobiology and oncology, the field currently faces vast mechanistic gaps. The precise molecular identity of microbial ligands, the spatial dynamics of microbial colonisation within TREM2+ macrophages, and the context‐dependent signalling networks governing this axis remain largely uncharted.

Along the systemic distal arm of this axis, the gut microbiota acts as a master rheostat calibrating baseline microglial and macrophage functional phenotypes. Commensal microbial metabolism generates a vast array of bioactive circulatory factors, including short‐chain fatty acids (SCFAs), secondary bile acids and lipidated bacterial membrane components.^87^ These metabolites continuously transit through systemic circulation, cross the blood–brain or blood–retinal barriers and fine‐tune microglial TREM2 expression and signalling threshold.^88^ In neurodegenerative proteinopathies such as AD and PD, intestinal dysbiosis—marked by the depletion of SCFAs‐producing commensals—triggers low‐grade systemic endotoxemia and a concurrent suppression of microglial TREM2 signalling tone. This deficit compromises microglial metabolic fitness, impairs Aβ and α‐synuclein aggregate compaction and perpetuates unresolving neuroinflammation.

At the systemic immunometabolic interface, the gut microbiota has recently emerged as an essential external rheostat that explains the marked heterogeneity and conflicting therapeutic outcomes observed in TREM2‐targeted cancer immunotherapy. In a groundbreaking investigation, Di Luccia et al. uncovered a reciprocal regulatory circuit between intestinal TREM2 signalling, commensal bacterial ecology and systemic anti‐tumour competence.^89^ Within the intestinal mucosa, TREM2 is constitutively expressed by resident lamina propria macrophages, where it actively dampens bactericidal activation and lipid sensing to maintain local immune tolerance.^90^ Crucially, targeted genetic deficiency or therapeutic antagonism of TREM2 in intestinal macrophages abolishes this immunosuppressive brake, initiating a metabolic and transcriptional rewiring that reshapes the luminal microbial architecture.^91^ This microbial restructuring enriches specific beneficial bacterial taxa that generate systemic immunomodulatory metabolites, including altered bile acid derivatives and SCFAs.^92^ Translocating through the mesenteric lymphatic and vascular circulation, these gut‐derived signals reprogram peripheral and distant TAMs towards a pro‐inflammatory, antigen‐presenting state, thereby relieving intra‐tumoural CD8+ T‐cell exhaustion and dramatically sensitising refractory solid tumours to anti‐PD‐1 ICB.^93^, ^94^ Faecal microbiota transplantation (FMT) and co‐housing experiments definitively proved that this enhanced anti‐tumour efficacy is microbially transmissible. These findings provide a compelling mechanistic explanation for the long‐standing discrepancies across preclinical cancer models, including GBM and melanoma.^95^ Because baseline microbiome compositions diverge substantially between commercial animal vendors and individual vivarium facilities, the net therapeutic outcome of TREM2 modulation is fundamentally conditioned by the recipient host's gut microbial baseline.^96^ Consequently, future clinical translation of TREM2 modulators must integrate metagenomic gut profiling as an indispensable companion biomarker to predict therapeutic response and reconcile conflicting clinical responses.^35^, ^97^

Locally, high‐dimensional STs and single‐cell profiling have revealed that solid tumours—including pancreatic, colorectal, hepatocellular and breast malignancies—harbour distinct, functional intra‐tumoural microbial niches.^98^ Intra‐tumoural bacteria do not merely exist as passive bystanders in necrotic cores; they are actively phagocytosed by and persist within TAMs, demonstrating a striking preferential enrichment inside TREM2+ lipid‐associated TAM subpopulations.^99^ Within these cells, intracellular bacterial pathogen‐associated molecular patterns (PAMPs) engage surface and endosomal pattern recognition receptors (PRRs), initiating complex signalling crosstalk with the canonical TREM2–DAP12–SYK axis. While classical PRR activation typically drives pro‐inflammatory anti‐tumour responses, co‐active TREM2 signalling acts as a metabolic circuit breaker—promoting FAO and LD accumulation that re‐channels microbially induced activation towards an immunosuppressive, tissue‐remodelling phenotype.^44^ Consequently, emerging paradigms hypothesise that intra‐tumoural microbes may functionally engage the TREM2 signalling hub to reinforce TAM‐mediated CD8+ T‐cell suppression, L‐arginine depletion via arginase‐1 (Arg1) upregulation and resistance to ICB. However, direct molecular evidence establishing a causal signalling link between bacterial PAMPs and cell‐autonomous TREM2–DAP12–SYK activation within the same macrophage remains preliminary and warrants validation in conditional models.^100^

Elucidating this systemic and niche‐specific microbial crosstalk opens transformative therapeutic opportunities for precision medicine. In CNS pathology, targeted restoration of gut eubiosis via postbiotics, SCFAs supplementation or FMT offers a non‐invasive approach to upregulate microglial TREM2, reinstate plaque encapsulation and attenuate neurodegeneration.^101^ In oncology, precise manipulation of the intra‐tumoural microbiome—utilising narrow‐spectrum narrow‐target antibiotics, engineered bacteriophages or microbial metabolite modulators—can re‐tune the PAMP/DAMP landscape within the tumour stroma. Disrupting the intra‐tumoural microbial drivers that sustain TREM2+ TAM polarisation promises to dismantle the immunosuppressive shield, restore CD8+ T‐cell infiltration and re‐sensitise cold tumours to anti‐PD‐1 or anti‐TREM2 immunotherapies. Bridging microbiome profiling with sTREM2 biomarker tracking will thus be crucial for stratifying patients eligible for combination chemo‐immunotherapy^102^ (Figure 5).

**FIGURE 5:**
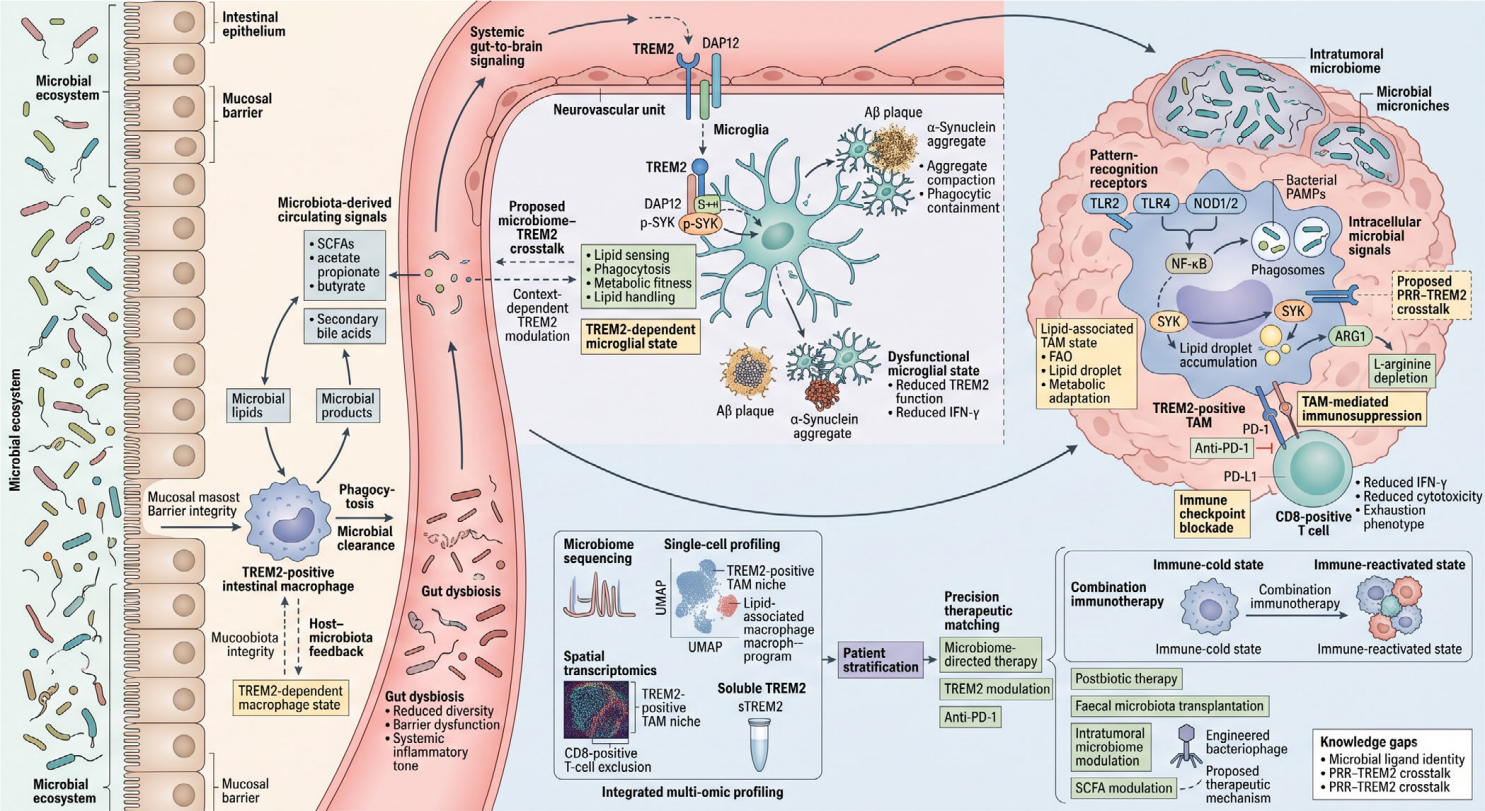
TREM2 as a candidate integrative myeloid signalling hub in the bidirectional gut–brain–tumour axis. (A) The systemic distal arm (gut–brain communication). Left: In the distal intestinal lumen, a balanced commensal microbiome maintains mucosal barrier integrity through gut‐resident TREM2+ macrophages, supporting microbial clearance and mucosal tissue homeostasis via parallel innate sensing pathways. Microbial‐derived circulating signals—including SCFAs, secondary bile acids and microbial lipid metabolites—enter the portal and systemic vasculature. Centre top: These systemic signals interact with the neurovascular unit at the BBB. Microglial TREM2 engages DAP12 and recruits phosphorylated SYK, sustaining a phenotypically competent microglial state defined by enhanced lipid sensing, phagocytosis, metabolic fitness and lipid handling. This state promotes compaction and phagocytic containment of neurodegenerative protein aggregates, such as Aβ plaques and α‐synuclein aggregates. Conversely, gut dysbiosis elevates systemic inflammatory tone, alters metabolite signatures and accelerates barrier breakdown, driving a dysfunctional microglial state characterised by impaired aggregate containment and neuroimmune dysregulation. (B) The local tumour arm (intra‐tumoural microbiome and TAM reprogramming). Right: Within the solid TME, low‐biomass intra‐tumoural bacteria occupy spatially restricted microniches and intracellular compartments (phagosomes) inside myeloid cells. Bacterial PAMPs engage PRRs to activate canonical NF‐κB signalling. Concurrently, TREM2–DAP12–SYK signalling in lipid‐associated TAMs orchestrates metabolic reprogramming, including heightened FAO and lipid droplet accumulation. Proposed PRR–TREM2 crosstalk drives immunosuppressive outputs, including Arg1 expression and local L‐arginine depletion. This immunosuppressive niche compromises neighbouring CD8+ cytotoxic T‐cell function, accelerating T‐cell exhaustion and mediating resistance to anti‐PD‐1 ICB. (C) Multi‐omic profiling, precision stratification and combinatorial therapeutics. Bottom: Integrated translational workflow combining stool and intra‐tumoural microbiome sequencing, spatial transcriptomics, scRNA‐seq and plasma/CSF sTREM2 biomarker quantification. Patient stratification guides precision therapeutic matching, transforming an immune‐cold tumour phenotype into an immune‐reactivated state through combination interventions: microbiome‐directed therapies, TREM2‐targeted modulation and anti‐PD‐1 checkpoint immunotherapy. Solid arrows represent established biological interactions; dashed arrows denote proposed crosstalk, context‐dependent signalling, candidate integration mechanisms or unresolved knowledge gaps. Aβ, amyloid‐β; Arg1, arginase 1; BBB, blood–brain barrier; CD8, cluster of differentiation 8; CSF, cerebrospinal fluid; DAP12, DNAX‐activating protein of 12 kDa; FAO, fatty acid oxidation; ICB, immune checkpoint blockade; NF‐κB, nuclear factor kappa‐light‐chain‐enhancer of activated B cells; PAMPs, pathogen‐associated molecular patterns; PD‐1, programmed cell death protein 1; PRRs, pattern‐recognition receptors; SCFAs, short‐chain fatty acids; scRNA‐seq, single‐cell RNA sequencing; sTREM2, soluble triggering receptor expressed on myeloid cells 2; SYK, spleen tyrosine kinase; TAM, tumour‐associated macrophage; TREM2, triggering receptor expressed on myeloid cells 2.

## 6 TREM2 IN CANCER

### 6.1 TREM2 in tumour‐associated myeloid cells

The TME comprises a complex and heterogeneous network of immune and stromal components, among which TAMs represent one of the most prevalent and functionally significant cell populations.^103^ TAMs critically shape tumour evolution by orchestrating immune dynamics, promoting growth and metastatic dissemination and modulating therapeutic responsiveness. TREM2 is preferentially expressed in TAMs and related myeloid subsets, where it serves as an immunoregulatory receptor that fine‐tunes cellular responses to damage‐associated cues within the TME. Engagement of TREM2 signalling can reprogram myeloid phenotypes, thereby shifting their functional balance between pro‐inflammatory and immunosuppressive states.^1^, ^8^ High‐dimensional scRNA‐seq and STs have fundamentally refined our understanding of TREM2+ myeloid architecture, mapping these cells to specific hypoxic, necrotic and lipid‐rich microdomains in both neurodegenerative lesions and tumour cores.^104^, ^105^ Rather than adhering to simplistic binary polarisation states, TREM2+ cells represent a distinct, highly specialised LAM or DAM phenotype.^106^ In primary CNS malignancies, particularly GBM, TREM2 signalling represents one of the most polarising controversies in cancer immunology, characterised by diametrically opposed findings across independent laboratories even when utilising identical syngeneic models. On one side of this debate, extensive investigations demonstrate that TREM2 exerts a tumour‐restraining, immunoprotective function. Seminal work by Zhong et al. revealed that TREM2 over‐expression on tumour‐associated myeloid cells suppresses intracranial glioma expansion and synergises with anti‐PD‐1 checkpoint blockade.^23^ Mechanistically, complementary studies by Zheng et al. and Peshoff et al. corroborated that TREM2 promotes microglial phagocytosis of viable and apoptotic glioma cells, enhances MHC‐II‐associated tumour antigen presentation to CD4+ T lymphocytes and compacts invasive tumour edges, whereas genetic ablation of TREM2 accelerates glioma progression and impairs T‐cell infiltration.^107^, ^108^ In sharp contrast to this protective paradigm, Sun et al. uncovered a potently immunosuppressive function of TREM2 within the same disease setting, demonstrating that glioma‐infiltrating TREM2+ myeloid cells actively blunt CD8+ T‐cell cytotoxicity; strikingly, both genetic deficiency and pharmacologic blockade of TREM2 triggered anti‐tumour immune reactivation, reduced tumour burden and significantly prolonged overall survival.^56^ This stark discrepancy prompted an editorial in *Neuro‐Oncology* to ask whether field observations distinguish true biological reality from experimental illusion, underscoring that TREM2 cannot be simply designated as an anti‐tumour receptor in the brain. Within these niches, TREM2 functions as a metabolic checkpoint coupling lipid sensing to downstream metabolic reprogramming. Ligand‐induced TREM2 activation fuels FAO and orchestrates LD biogenesis and turnover.^105^ This bioenergetic adaptation prevents lipotoxicity, preserves mitochondrial bioenergetics and sustains the phagocytic and degradative vigour of myeloid cells under pathological stress.^34^

This phenotypic dichotomy in GBM is critically governed by four interconnected biological and experimental determinants: myeloid cell ontogeny, treatment‐induced niche remodelling, host microbiome composition and spatial lipid architecture. First, myeloid ontogeny dictates distinct functional programs: while yolk sac‐derived resident microglia initially co‐opt TREM2 for immune surveillance and phagocytic clearance of apoptotic debris, bone marrow‐derived monocyte macrophages (Mo‐TAMs) recruited via disrupted blood–tumour barriers frequently repurpose TREM2 to reinforce wound‐healing, pro‐angiogenic and immunosuppressive programs.^3^, ^109^ Crucially, as recently elucidated by Kloosterman et al., this ontogenetic balance dynamically shifts following standard clinical interventions: in post‐treatment and recurrent GBM, radiotherapy and alkylating chemotherapy trigger a massive influx of circulating monocyte‐derived macrophages that massively outnumber resident microglia, wherein TREM2‐expressing Mo‐TAMs actively recycle myelin and cellular lipids to fuel GBM malignancy and therapeutic resistance.^99^ Second, the host gut microbiome acts as an extrinsic immune rheostat that calibrates myeloid responsiveness. As demonstrated by Di Luccia et al., variations in intestinal microbiota composition between animal facilities dictate baseline myeloid metabolic states and profoundly modulate therapeutic responsiveness to TREM2 blockade, potentially explaining divergent survival outcomes across ostensibly identical murine cohorts.^89^, ^96^ Third, intra‐tumoural spatial heterogeneity establishes contrasting microenvironments: within necrotic, hypoxic tumour cores, excessive myelin and lipid uptake drives TREM2‐dependent foam cell formation and metabolic exhaustion, paralyzing anti‐tumour activity; conversely, at infiltrative peritumoural margins, moderate TREM2 activation preserves microglial homeostatic compaction and limits excitotoxic neuronal damage.^3^, ^110^ Consequently, rather than functioning as a universal tumour suppressor, TREM2 operates along a highly plastic continuum whose net immunotherapeutic outcome is dictated by the precise balance of microglial versus monocyte‐derived macrophage infiltration, the treatment history of the tumour and systemic metabolic cues.

Crucially, the functional identity of TREM2+ myeloid cells in GBM is not static, but undergoes profound spatiotemporal remodelling following standard clinical interventions.^111^ While treatment‐naïve primary gliomas are dominated by yolk sac‐derived resident microglia (Mg‐TAMs) that can mediate homeostatic surveillance and apoptotic clearance, therapy‐induced tissue injury drastically alters this balance. In a landmark study, Kloosterman et al. demonstrated that standard radiotherapy and chemotherapeutic stress induce severe blood–tumour barrier permeability, extensive demyelination and widespread cellular senescence, which collectively mobilise a massive influx of bone marrow‐derived circulating monocytes into the recurrent tumour niche.^99^ In post‐treatment and recurrent GBM, these monocyte‐derived macrophages (Mo‐TAMs) rapidly outcompete resident microglia, becoming the predominant myeloid population and displaying robust upregulation of TREM2 alongside lipid‐handling receptors.^112^

Rather than executing protective tumour phagocytosis, these post‐treatment TREM2+ Mo‐TAMs co‐opt the receptor's lipid‐sensing machinery to drive ‘macrophage‐mediated myelin recycling’.^99^ Through the TREM2–DAP12 axis, infiltrating Mo‐TAMs efficiently ingest massive quantities of myelin debris and damaged lipid aggregates shed from dying oligodendrocytes and irradiated brain parenchyma. Instead of neutralising these lipids, TREM2 signalling accelerates endolysosomal cholesterol trafficking and fatty acid mobilisation, converting Mo‐TAMs into lipid‐laden ‘foam cells’ that transfer bioactive metabolic precursors to neighbouring GBM cells.^113^ This TREM2‐dependent lipid scavenging and trophic transfer directly fuels GBM cell survival, stimulates adaptive resistance to irradiation and preserves glioma stem‐like cell self‐renewal.^114^ Consequently, the rapid influx and metabolic hijacking of TREM2‐expressing Mo‐TAMs in recurrent GBM completely inverts the therapeutic paradigm, providing a definitive molecular rationale for deploying TREM2‐blocking or lipid‐depleting strategies specifically in post‐treatment disease settings.^115^, ^116^

Conversely, investigations in peripheral solid malignancies—including hepatocellular carcinoma (HCC), colorectal cancer and melanoma—indicate a divergent functional role for TREM2.^23^, ^56^ In these settings, TREM2 is primarily expressed by TAMs originating from circulating monocytes rather than by CNS‐resident microglia.^56^ Within this context, TREM2 signalling drives an immunosuppressive, tumour‐promoting program marked by elevated IL‐10 production, diminished antigen‐presenting capacity and a metabolic shift towards oxidative phosphorylation.^45^ Consistently, genetic ablation or therapeutic blockade of TREM2 in murine melanoma and liver cancer models enhances CD8^+^ T‐cell infiltration and cooperates with PD‐1 or CTLA‐4 checkpoint inhibition, highlighting TREM2 as a key brake on anti‐tumour immunity outside the CNS. Conversely, in peripheral solid tumours, TREM2 reinforces immune suppression and tumour tolerance. Consequently, therapeutic interventions in these settings must focus on inhibiting or blocking TREM2 signalling to unleash anti‐tumour immunity.^3^

### 6.2 Mechanisms of TREM2 signalling in cancer

The downstream signalling architecture of TREM2 is central to defining its functional impact within the TME. Following ligand engagement, TREM2 associates with adaptor molecules such as DAP12 and DAP10, thereby triggering a series of intracellular signalling cascades.^117^, ^118^ DAP12 facilitates recruitment and activation of SYK, thereby modulating downstream immune signalling networks, whereas DAP10 engages PI3K pathways that support cellular survival, proliferative capacity and immunoregulatory functions.^117^, ^118^ These signalling pathways are essential for the proper functioning of myeloid cells in the TME and can influence the balance between immune activation and suppression (Figure 3).

In cancers, the ligands of TREM2 are thought to include various damage‐associated molecular patterns (DAMPs) and lipids that are released from dying tumour cells, necrotic tissue or damaged extracellular matrix components.^22^ One of the key classes of TREM2 ligands in cancer is sphingolipids, which are enriched in the CNS and have been shown to bind TREM2 on myeloid cells, particularly in GBM.^22^ A prominent molecular driver of TREM2‐mediated immunosuppression in peripheral malignancies is the TREM2–ApoE signalling axis.^119^ ApoE functions as a bona fide, high‐affinity extracellular ligand for the TREM2 receptor. Within the lipid‐rich TME, both dying cancer cells and surrounding myeloid populations abundantly secrete ApoE. Extracellular ApoE engages the positively charged basic surface of the TREM2 IgV domain, inducing receptor multimerisation and robust phosphorylation of the DAP12 and DAP10 adaptor complexes. Downstream activation of the SYK–PI3K–mTOR signalling pathway instructs TAMs to acquire a distinct ApoE/TREM2 co‐expressing lipid‐associated macrophage phenotype.^120^ This engagement enhances FAO, drives intracellular LD accumulation and stimulates the transcriptional upregulation of anti‐inflammatory cytokines, including TGF‐β and IL‐10. Concurrently, the TREM2–ApoE cascade promotes high expression of Arg1, resulting in severe local depletion of extracellular L‐arginine. Because L‐arginine is indispensable for T‐cell receptor expression and cytotoxic function, this metabolic alterations effectively paralyses tumour‐infiltrating CD8+ T cells and drives resistance to ICB.^121^ Disrupting the physical TREM2–ApoE interaction or targeting this downstream lipid‐metabolic cascade therefore represents a compelling immunotherapeutic strategy to dismantle the tumour‐supportive myeloid niche.^45^

Beyond immune regulation, recent evidence suggests that TREM2 may also mitigate neuronal excitotoxicity by promoting the clearance of glutamate‐damaged neurons and enhancing the expression of glutamate transporters in microglia.^122^, ^123^ Microglial TREM2 facilitates the clearance of apoptotic neurons damaged by glutamate excitotoxicity, thereby limiting secondary neurodegeneration.^124^ The interaction between TREM2 and sphingolipids in the TME has been shown to activate a protective signalling response in TAMs, leading to a shift towards a more anti‐inflammatory phenotype.^125^ This reprogramming of TAMs by TREM2 ligands may contribute to the immunoprotective role of TREM2 in CNS cancers, as it enhances the phagocytic activity of myeloid cells and promotes the clearance of tumour debris. However, in peripheral cancers, the same pathways may drive the immunosuppressive polarisation of TAMs. Mechanistically, TREM2 signalling promotes fatty acid uptake and oxidative metabolism in TAMs, enhances expression of Arg1 and IL‐10, and suppresses antigen presentation, collectively contributing to T‐cell dysfunction within the TME. In the peripheral TME, TREM2 signalling facilitates immunosuppression through metabolic and epigenetic reprogramming. TREM2 engagement promotes the expression of fatty acid transporters and enhances FAO, which is a metabolic hallmark of pro‐tumoural, immunosuppressive macrophage programs. This metabolic shift is linked to the increased production of anti‐inflammatory metabolites and the induction of Arg1.

The biological equilibrium between membrane‐bound TREM2 and its proteolytically cleaved sTREM2 constitutes a pivotal regulatory axis that governs immune phenotypes across tissues.^126^ Membrane‐bound TREM2 is constitutively cleaved at the His157‐Ser158 peptide bond by the zinc‐dependent metalloproteases ADAM10/17 (A Disintegrin And Metalloprotease 10/17), releasing the soluble extracellular IgV domain into the interstitial and systemic fluids. While sTREM2 has traditionally been interpreted as a passive liquid biomarker mirroring myeloid cell density and activation in NDDs and peripheral cancers, emerging functional evidence establishes sTREM2 as an active, bio‐potent paracrine mediator.^45^ Within the TME, robust ADAM10/17‐mediated ectodomain shedding reduces the density of full‐length signalling receptors on TAMs, while simultaneously generating high concentrations of sTREM2 in the stromal fluid. Once shed, extracellular sTREM2 can bind to cell‐surface receptors on neighbouring myeloid and malignant cells, triggering trans‐activation of the PI3K‐AKT and NF‐κB signalling pathways in a cell‐autonomous and paracrine manner.^45^ This paracrine cascade reinforces macrophage immunosuppressive polarisation, stimulates the secretion of immunosuppressive cytokines such as IL‐10 and TGF‐β, promotes tumour cell survival and accelerates therapeutic resistance against ICB. Consequently, ADAM10/17‐mediated shedding serves not merely as a receptor down‐regulation mechanism, but as an active metabolic switch that amplifies tumour‐promoting immunosuppressive circuits throughout the microenvironment.^127^

However, the pro‐tumourigenic role of TREM2 in peripheral malignancies is not universal, and accumulating evidence demonstrates that TREM2 can exert anti‐tumour or protective functions in specific peripheral tumour contexts.^45^ A notable example is HCC, where TREM2 expressed on tissue‐resident Kupffer cells and infiltrating macrophages plays a multifactorial tumour‐suppressive role. In hepatic tumour models, TREM2 deficiency dampens apoptotic cell clearance, exacerbates chronic liver injury and accelerates hepatocarcinogenesis, whereas intact TREM2 signalling restrains tumour initiation by preserving tissue homeostasis and restricting oncogenic inflammation.^128^ Similarly, in colorectal cancer and specific gastrointestinal stromal models, TREM2+ macrophages can facilitate the clearance of damaged epithelial cells and limit dysbiosis‐driven tumourigenesis.^90^ These contrasting preclinical observations underscore that TREM2 activity in peripheral oncology is tightly governed by the underlying organ architecture, aetiology and local stromal cytokine milieu, rather than acting as a simple, uniform driver of immune suppression.^102^

## 7 CONTEXT‐DEPENDENT THERAPEUTIC STRATEGIES FOR TARGETING TREM2

TREM2 has emerged as a central regulator of immune dynamics, with broad implications across diverse pathological contexts, including NDDs, malignancies, autoimmune diseases and cerebrovascular conditions.^5^, ^12^, ^122^, ^129^, ^130^, ^131^, ^132^ Although TREM2 is increasingly acknowledged as a critical immunoregulatory node, translating its biological promise into therapeutic applications remains complex. Its context‐dependent effects and multi‐layered signalling networks pose significant challenges for rational intervention. As our understanding of TREM2 biology continues to deepen, careful consideration of unresolved mechanistic questions and strategic research priorities will be essential to unlock its full therapeutic potential across diverse disease settings (Table 1).

**TABLE 1:** Context‐dependent roles and therapeutic implications of TREM2 across diverse disease landscapes.

Disease	Specific disease	Primary cell type	Key ligands and mechanisms	Pathological outcome	Therapeutic strategy
**NDDs**	**AD**	Microglia (DAM phenotype)	**Ligands**: Aβ oligomers, ApoE–lipid complexes, anionic lipids. **Mechanism**: DAP12–SYK–mTOR signalling drives transition to DAM state; facilitates phagocytosis and lipid metabolism.^1^, ^3^	**Protective**: Promotes amyloid plaque compaction; forms microglial barrier to limit neuritic dystrophy.^4^, ^5^	**Agonism**: Agonistic Abs (e.g., AL002) to cross‐link TREM2, stabilise surface expression and boost microglial survival/activity.^6^, ^7^
	**PD**	Microglia	**Ligands**: α‐synuclein aggregates, lipids. **Mechanism**: Regulates inflammation and clearance of protein aggregates.^8^	**Protective**: Facilitates clearance of α‐synuclein; reduces neuroinflammation.^8^, ^9^	**Agonism**: Enhancing TREM2 signalling to promote aggregate clearance.^9^
	**FTD/ALS**	Microglia	**Ligands**: TDP‐43, apoptotic neurons. **Mechanism**: Sensing of NAMPs.^10^	**Protective**: Clears TDP‐43 and apoptotic debris; slows neurodegeneration.^10^, ^11^	**Agonism**: Gene therapy or agonists to restore microglial function.^10^, ^12^
**CNS malignancies**	**GBM**	Microglia/CNS‐associated Macrophages	**Ligands**: CNS‐enriched sphingolipids, necrotic debris. **Mechanism**: Supports phagocytosis and metabolic fitness in the hypoxic TME.^13^, ^14^	**Protective/Anti‐tumour**: Facilitates immune clearance of tumour debris; restrains tumour progression.^13^	**Agonism/Restoration**: Restoring TREM2 expression (e.g., via AAV) synergises with anti‐PD‐1 therapy.^13^, ^14^
**Peripheral cancer**	**Peripheral solid tumours**	TAMs	**Ligands**: Tumour‐derived lipids, DAMPs. **Mechanism**: Drives immune‐suppressive polarisation; promotes IL‐10/TGF‐β release.^15^, ^20^	**Detrimental/pro‐tumour**: Reinforces immune suppression; inhibits CD8+ T‐cell infiltration and function.^15^	**Antagonism/Blockade**: Blocking Abs or depleting agents to disrupt immunosuppressive TAMs and unleash T‐cell immunity.^20^, ^21^
**Cerebrovascular**	**Ischaemic stroke**	Microglia	**Ligands**: Apoptotic neuronal debris. **Mechanism**: Regulates inflammation resolution and phagocytosis.^16^, ^17^	**Protective**: Enhances neuroprotection and functional recovery post‐ischemia.^17^, ^18^	**Agonism**: Approaches to enhance microglial phagocytosis and inflammation resolution.^18^

Abbreviations: AAV, adeno‐associated virus; Abs, antibodies; AD, Alzheimer's disease; ALS, amyotrophic lateral sclerosis; ApoE, apolipoprotein E; Aβ, amyloid‐β; CD8, cluster of differentiation 8; CNS, central nervous system; DAM, disease‐associated microglia; DAMPs, damage‐associated molecular patterns; DAP12, DNAX‐activating protein of 12 kDa; FTD, frontotemporal dementia; GBM, glioblastoma; IL‐10, interleukin‐10; mTOR, mechanistic target of rapamycin; NAMPs, neuronal damage‐associated molecular patterns; NDDs, neurodegenerative diseases; PD, Parkinson's disease; PD‐1, programmed cell death protein 1; SYK, spleen tyrosine kinase; TAMs, tumour‐associated macrophages; TDP‐43, TAR DNA‐binding protein 43; TGF‐β, transforming growth factor‐β; TME, tumour microenvironment; TREM2, triggering receptor expressed on myeloid cells 2.

### 7.1 Therapeutic targeting of TREM2 in cancer

Owing to its central function in shaping immune dynamics within tumours, TREM2 has gained attention as a candidate target for cancer immunotherapy. A range of therapeutic approaches are currently under investigation to manipulate TREM2 activity in the TME, including agonistic and antagonistic antibodies, as well as combinatorial strategies with ICB.^9^, ^55^, ^89^ Notably, combinatorial regimens integrating TREM2‐directed interventions with ICB have demonstrated encouraging outcomes in preclinical models. Strategic modulation of TREM2 signalling in TAMs may facilitate reprogramming of the TME from an immunosuppressive niche towards a more immunostimulatory landscape, thereby enhancing responsiveness to established immunotherapeutic modalities (Figure 6).

**FIGURE 6:**
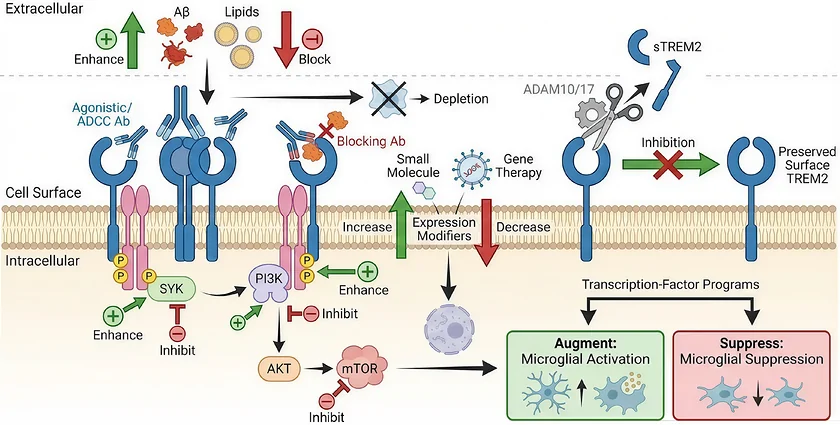
Strategic points of intervention for modulating TREM2 signalling. Therapeutic targeting of the TREM2 axis can be approached at multiple regulatory levels. Extracellular strategies include: (i) ligand modulation—either enhancing or limiting ligand availability and affinity to fine‐tune receptor engagement; (ii) agonistic or ADCC‐enabled antibodies designed to cluster TREM2 for signal amplification or to mediate selective cell depletion; and (iii) regulation of ectodomain shedding, whereby inhibition of metalloproteases such as ADAM10/17 reduces receptor cleavage and preserves membrane‐bound TREM2. At the receptor and cellular level, expression‐modifying approaches—such as small‐molecule regulators or gene‐based therapies—can increase or decrease TREM2 abundance. Intracellularly, pathway‐specific modulators may potentiate or suppress DAP10/12–SYK–PI3K signalling networks, thereby adjusting the magnitude and persistence of downstream responses. These layered inputs ultimately converge on transcriptional programs that govern TREM2‐dependent gene expression, shaping microglial activation states and effector phenotypes. ADAM10/17, a disintegrin and metalloproteinase 10/17; ADCC, antibody‐dependent cellular cytotoxicity; DAP10, DNAX‐activating protein of 10 kDa; DAP12, DNAX‐activating protein of 12 kDa; PI3K, phosphoinositide 3‐kinase; SYK, spleen tyrosine kinase; TREM2, triggering receptor expressed on myeloid cells 2.

For instance, in GBM, where TREM2 expression declines in TAMs as the disease progresses, reconstitution of TREM2 through adeno‐associated virus (AAV)‐based gene delivery has been reported to inhibit tumour expansion and potentiate anti‐tumour immune activity.^23^ Notably, this strategy exhibits synergistic effects when combined with anti‐PD‐1 blockade, underscoring the therapeutic rationale for integrating TREM2‐directed interventions with immune checkpoint inhibitors to counteract tumour‐driven immune evasion.^3^, ^133^

While TREM2 has been primarily studied in the context of NDDs, its role in cancer is becoming increasingly recognised. Targeting TREM2 in the TME offers a promising strategy to modulate myeloid cell activity and enhance the effectiveness of cancer immunotherapies. However, the role of TREM2 in cancer is complex, and its effects can vary depending on the tumour type and the specific immune context. It is crucial to better understand the specific mechanisms that govern TREM2 signalling in different cancers and how it interacts with other immune regulatory pathways. Future research should focus on elucidating the precise mechanisms by which TREM2 regulates immune responses in cancer and developing therapeutic strategies that can exploit its dual roles in both immunosuppression and immune activation. The organ‐specific functions of TREM2, as well as its interaction with various ligands in the TME, will likely be key factors in determining the success of TREM2‐based therapies in cancer treatment.

The expanding recognition of TREM2 in oncology—particularly its influence on TAMs—introduces additional complexity to its biological landscape. TREM2 serves as a central determinant of macrophage polarisation and has been linked to tumour advancement, immune escape mechanisms and modulation of immunotherapeutic responses. In peripheral solid malignancies, including melanoma, colorectal carcinoma and HCC, TREM2 expression is enriched within immunosuppressive, metabolically reprogrammed TAM populations.^132^, ^134^, ^135^ Conversely, in peripheral solid tumours, the goal is often to inhibit TREM2‐mediated immunosuppression. Here, antagonistic or blocking antibodies are employed to disrupt the maintenance of immunosuppressive TAMs, thereby unleashing T‐cell responses and enhancing the efficacy of immune checkpoint inhibitors.^9^ This strategy functions as a myeloid checkpoint blockade, reducing the number of immunosuppressive TREM2+ TAMs and promoting anti‐tumour immunity.^9^, ^136^ Combination regimens of anti‐TREM2 with immune checkpoint inhibitors such as anti‐PD‐1 or anti‐CTLA‐4 have shown additive or synergistic effects in several preclinical models, further supporting the concept that TREM2 can be therapeutically relieved to reprogram the myeloid compartment and potentiate immunotherapy.^55^, ^137^, ^138^, ^139^ In this context, TREM2‐targeting antibodies generally act as antagonists or depleting agents, in clear contrast to the agonistic designs being explored in AD.

In contrast, the consequences of TREM2 signalling in GBM and other CNS tumours appear more nuanced and highly context‐dependent. In these settings, TREM2 is primarily enriched within microglia‐dominated myeloid populations, and accumulating evidence suggests a predominantly immunoprotective rather than purely immunosuppressive function. In murine GBM models, genetic ablation or pharmacologic inhibition of TREM2 accelerates tumour progression, promotes a shift of TAMs towards an Arg1^+^ immunosuppressive phenotype, and exacerbates T‐cell dysfunction. Conversely, enforced TREM2 expression or AAV–mediated delivery to CNS‐resident myeloid cells suppresses tumour growth and enhances responsiveness to anti‐PD‐1 immunotherapy.^23^ Thus, in the brain tumour setting, the therapeutic directionality of TREM2 modulation remains an open question subject to active debate. While agonistic approaches or gene‐delivery platforms aimed at enhancing microglial phagocytosis and MHC‐II presentation show therapeutic promise in treatment‐naïve, microglia‐dominated preclinical models, the pronounced accumulation of immunosuppressive, myelin‐recycling TREM2+ monocyte‐derived macrophages in post‐treatment recurrent GBM strongly supports the deployment of TREM2 antagonists or depleting antibodies to break tumour tolerance.^56^, ^99^ These divergent mechanistic pathways indicate that TREM2‐targeted therapies in neuro‐oncology cannot follow a uniform regimen, but instead demand rigorous patient stratification based on prior treatment status, myeloid ontogeny composition and intra‐tumoural lipid burden.

Nevertheless, therapeutic targeting of TREM2 in oncology is accompanied by significant challenges. The pronounced heterogeneity of TAMs across malignancies and tissue compartments suggests that the functional consequences of TREM2 signalling are likely to be highly context‐specific. As such, indiscriminate modulation of TREM2 may yield unintended effects, including adaptive resistance, heightened inflammatory toxicity or off‐target immune perturbations. An approach that proves advantageous in peripheral solid tumours could be counterproductive in CNS cancers, and vice versa.

### 7.2 Targeting TREM2 in NDDs

Genetic and functional evidence provides a strong rationale for TREM2 agonism in AD: restoring or enhancing TREM2 signalling may help normalise microglial function and promote the clearance of protein aggregates and lipid debris.

In the context of AD, therapeutic strategies primarily focus on TREM2 agonism. Agonistic antibodies, such as AL002, are designed to cross‐link TREM2, stabilising it on the cell surface and enhancing downstream DAP12 signalling to boost microglial survival and phagocytosis.^29^ In several AD mouse models, TREM2 agonist antibodies have been reported to reduce amyloid burden, increase plaque compaction, attenuate neuritic dystrophy and partially preserve cognitive performance.

These preclinical data have led to the development of humanised TREM2‐targeting antibodies that are now in early‐phase clinical testing in prodromal or mild AD. First‐in‐human studies indicate acceptable safety and pharmacokinetic profiles, with robust target engagement on circulating and CNS‐resident myeloid cells.^140^ Pharmacodynamic readouts from some programs include transient increases in CSF sTREM2, shifts in microglial‐related biomarkers and modulation of neuroinflammatory signatures, consistent with in vivo activation of TREM2‐dependent pathways.^141^ However, it remains unclear whether sustained TREM2 agonism will translate into clinically meaningful benefits on cognition or long‐term disease progression. Key challenges include efficient BBB penetration, optimal dosing to avoid over‐activation of microglia and the possibility that the therapeutic window is restricted to early disease stages before extensive neuronal loss and tau pathology have occurred.

At the same time, therapeutic strategies aimed at modulating TREM2 activity must reckon with the functional variability introduced by different TREM2 variants and disease contexts 58. While boosting TREM2 activity in carriers of loss‐of‐function mutations such as R47H may be beneficial, excessive or prolonged activation could theoretically drive chronic inflammation or exacerbate other pathologies. Future approaches are therefore likely to focus on stage‐specific and context‐dependent enhancement of TREM2 signalling, particularly in early‐stage neurodegeneration, where its activation might facilitate the clearance of toxic proteins such as Aβ and tau. Importantly, the role of TREM2 in tau‐related pathology remains controversial: some studies suggest that TREM2 over‐expression or hyperactivation can worsen tau‐induced neurodegeneration, whereas others report protective effects.^142^ Equally important is the re‐evaluation of TREM2 agonism in CNS pathologies, where therapeutic activation may represent a double‐edged sword depending on disease staging.^143^ Although TREM2 agonism demonstrates clear neuroprotective benefits during early amyloid‐β seeding by driving plaque compaction and microglial survival, evidence from late‐stage neurodegeneration and pure tauopathy models paints a far more nuanced picture.^73^ In advanced stages of AD or tau‐driven neurodegeneration, sustained TREM2 hyperactivation or prolonged over‐expression can paradoxically aggravate neuroinflammation, accelerate tau seeding and spreading across synaptic networks, and worsen cortical atrophy.^74^ Under chronic pathological stress, microglial metabolic fitness becomes exhausted, converting TREM2‐driven activation into a source of unresolving, neurotoxic inflammatory signalling.^74^ Therefore, indiscriminate enhancement of TREM2 signalling across all disease stages carries genuine risks of disease exacerbation. Thus, a more precise understanding of the temporal, genetic and microenvironmental factors that shape TREM2 function will be essential for designing TREM2‐targeted therapies that maximise neuroprotection while minimising potential harm.

Moreover, effective deployment of TREM2 agonists or antagonists will likely depend on delivery platforms capable of selectively engaging tumour‐associated myeloid subsets while preserving homeostatic macrophage populations in non‐tumour tissues. Moving forward, research efforts should prioritise mechanistic dissection of TREM2's context‐dependent roles within distinct tumour ecosystems, clarify its crosstalk with other immune checkpoint pathways and metabolic circuits, and develop biomarker‐informed frameworks to identify patients and tumour contexts most amenable to TREM2‐targeted interventions (Figure 7). It should be noted that current research on TREM2 in CNS malignancies is predominantly restricted to GBM. Whether similar immunoprotective mechanisms apply to other brain tumours, such as medulloblastoma or meningioma, remains an open question for future investigation.

**FIGURE 7:**
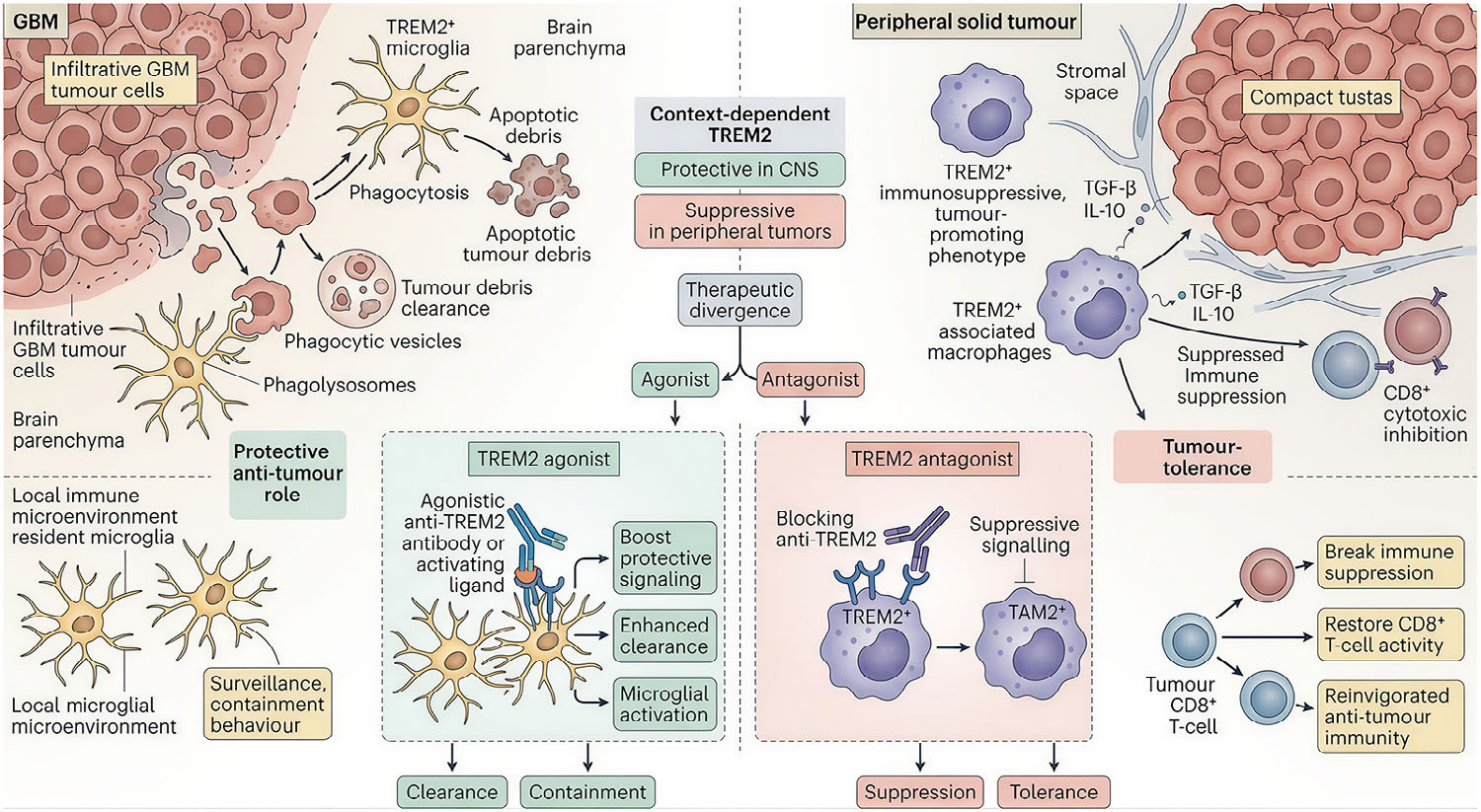
The context‐dependent dichotomy of TREM2 function in cancer. This diagram illustrates the contrasting biological roles of TREM2 signalling in CNS malignancies versus peripheral solid tumours, dictating opposing therapeutic strategies. (Left) In the CNS microenvironment (e.g., GBM), TREM2‐expressing microglia exert a protective, anti‐tumour role. TREM2 signalling facilitates the phagocytosis and clearance of tumour cells and debris, leading to tumour containment. Consequently, therapeutic strategies in this context aim to employ TREM2 agonists to boost this protective function. (Right) Conversely, in the peripheral TME of solid tumours (e.g., liver cancer, melanoma), TREM2 signalling in TAMs fosters a tumour‐promoting, immunosuppressive role. TREM2 activation drives TAMs to release immunosuppressive factors, such as TGF‐β and IL‐10, which inhibit cytotoxic CD8+ T‐cell responses and reinforce tumour tolerance. Therefore, therapeutic interventions in peripheral cancers require TREM2 antagonists to break immune suppression. CD8, cluster of differentiation 8; CNS, central nervous system; GBM, glioblastoma; IL‐10, interleukin‐10; TAMs, tumour‐associated macrophages; TGF‐β, transforming growth factor‐β; TME, tumour microenvironment; TREM2, triggering receptor expressed on myeloid cells 2.

### 7.3 The need for specificity in therapeutic targeting

A central obstacle in the development of TREM2‐directed therapeutics is achieving sufficient target specificity. TREM2 participates in multiple fundamental immune processes—including phagocytosis, cytokine regulation and survival signalling—raising the possibility that broad modulation may disrupt immune equilibrium or impair tissue repair mechanisms. Consequently, therapeutic strategies must prioritise the design of highly selective agonists or antagonists capable of fine‐tuning TREM2 activity while minimising risks of excessive inflammation or unintended immunosuppression.^31^, ^60^

Moreover, the pleiotropic roles of TREM2 across pathological conditions demand context‐tailored therapeutic frameworks. In NDDs, interventions may aim to potentiate TREM2‐driven clearance of toxic aggregates and restore microglial homeostasis, whereas in oncology, strategies are more likely to concentrate on reshaping the immune landscape within the TME. Delineating these disease‐specific functional programs will be essential for the rational design of precision therapies capable of calibrating TREM2 activity to the distinct biological demands of each clinical setting.

Within the framework of personalised medicine, therapeutic strategies must be dichotomised: local delivery of TREM2 agonists may benefit CNS indications (AD, GBM), whereas systemic administration of TREM2 antagonists could be prioritised for peripheral solid tumours to avoid off‐target activation of suppressive TAMs.^144^ Future research should prioritise elucidating the molecular mechanisms underlying TREM2's diverse functions and exploring context‐dependent strategies for targeting this receptor. Through these efforts, we can fully harness the therapeutic potential of TREM2, providing novel and promising avenues for the treatment of patients afflicted with a spectrum of debilitating diseases (Figure 8).

**FIGURE 8:**
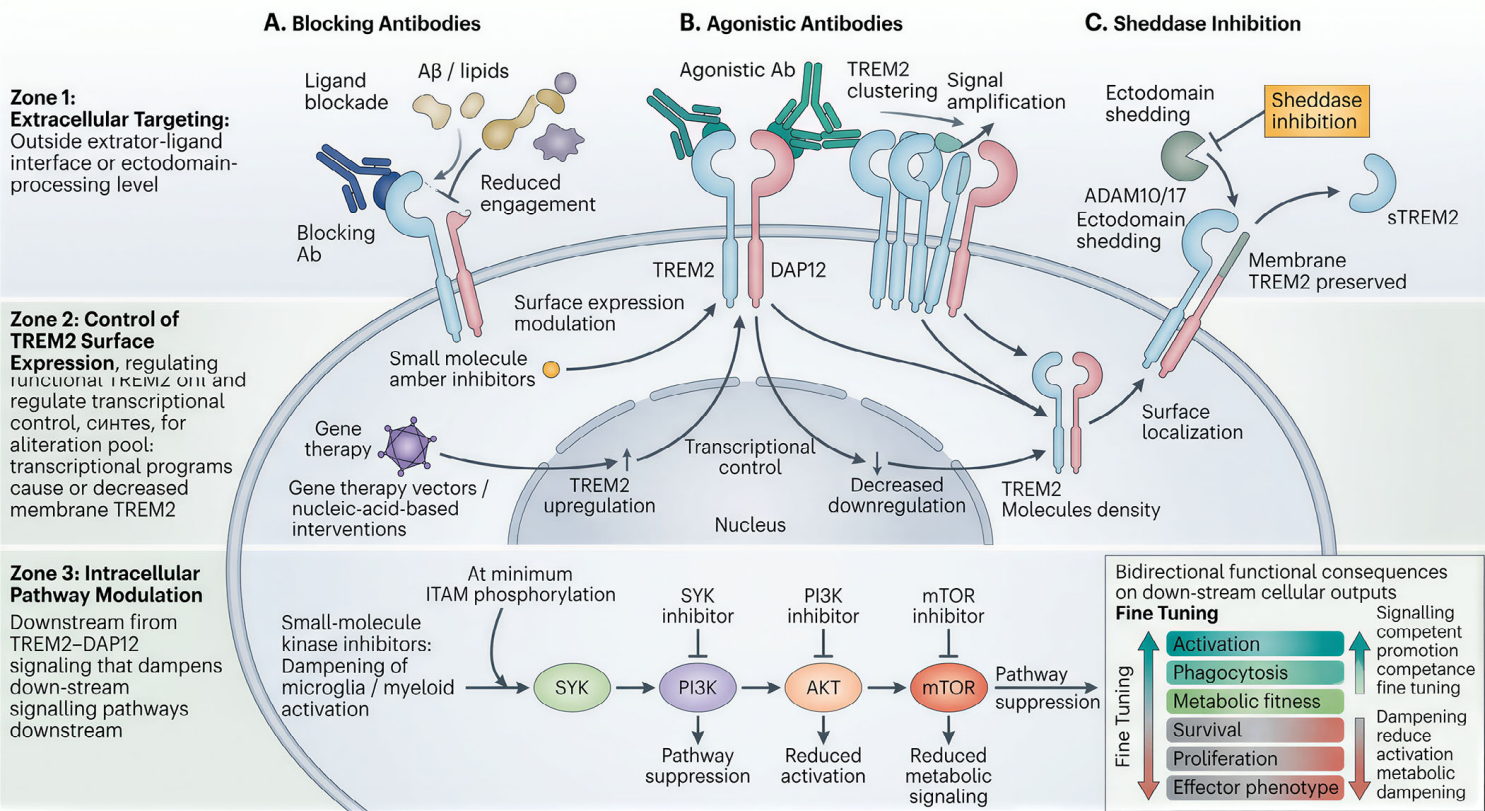
Therapeutic strategies targeting the TREM2 signalling pathway. This diagram illustrates diverse therapeutic approaches designed to modulate TREM2 function, categorised by their site of action into three zones. (Zone 1) Extracellular Targeting: Interventions aimed at the receptor‐ligand interface or ectodomain. Blocking Abs bind to the TREM2 ligand‐binding domain, preventing interaction with ligands such as Aβ species and lipids. Conversely, Agonistic Abs cross‐link multiple TREM2 receptors to promote clustering and boost downstream signalling. Sheddase inhibition involves blocking ADAM10/17 proteases, which prevents the proteolytic cleavage of surface TREM2, thereby maintaining receptor density. (Zone 2) Surface Expression Modulation: Strategies to regulate the total pool of functional TREM2 receptors on the cell surface. Agents such as small molecules or gene therapy vectors enter the cell to target nuclear transcription, leading to either an increase or decrease in overall TREM2 expression and surface localisation. (Zone 3) Intracellular Pathway Modulation: Small molecule kinase inhibitors are employed to block specific nodes within the downstream signalling cascade initiated by DAP12 ITAM phosphorylation. Inhibitors targeting SYK, PI3K or mTOR can dampen the intracellular signals responsible for microglial activation and metabolic regulation. Aβ, amyloid‐β; Abs, antibodies; ADAM10/17, a disintegrin and metalloproteinase 10/17; DAP12, DNAX‐activating protein of 12 kDa; ITAM, immunoreceptor tyrosine‐based activation motif; mTOR, mechanistic target of rapamycin; PI3K, phosphoinositide 3‐kinase; SYK, spleen tyrosine kinase; TREM2, triggering receptor expressed on myeloid cells 2.

## 8 TRANSLATIONAL BARRIERS AND THERAPEUTIC OPTIMISATION

Despite the compelling preclinical rationale positioning TREM2 as a master regulator of myeloid cell plasticity, translating these biological insights into effective clinical interventions faces a myriad of formidable pharmacodynamic, pharmacokinetic and immunological hurdles.^65^ A critical evaluation of these translational barriers is essential to guide the next generation of TREM2‐targeted precision therapeutics (Figure 9).

**FIGURE 9:**
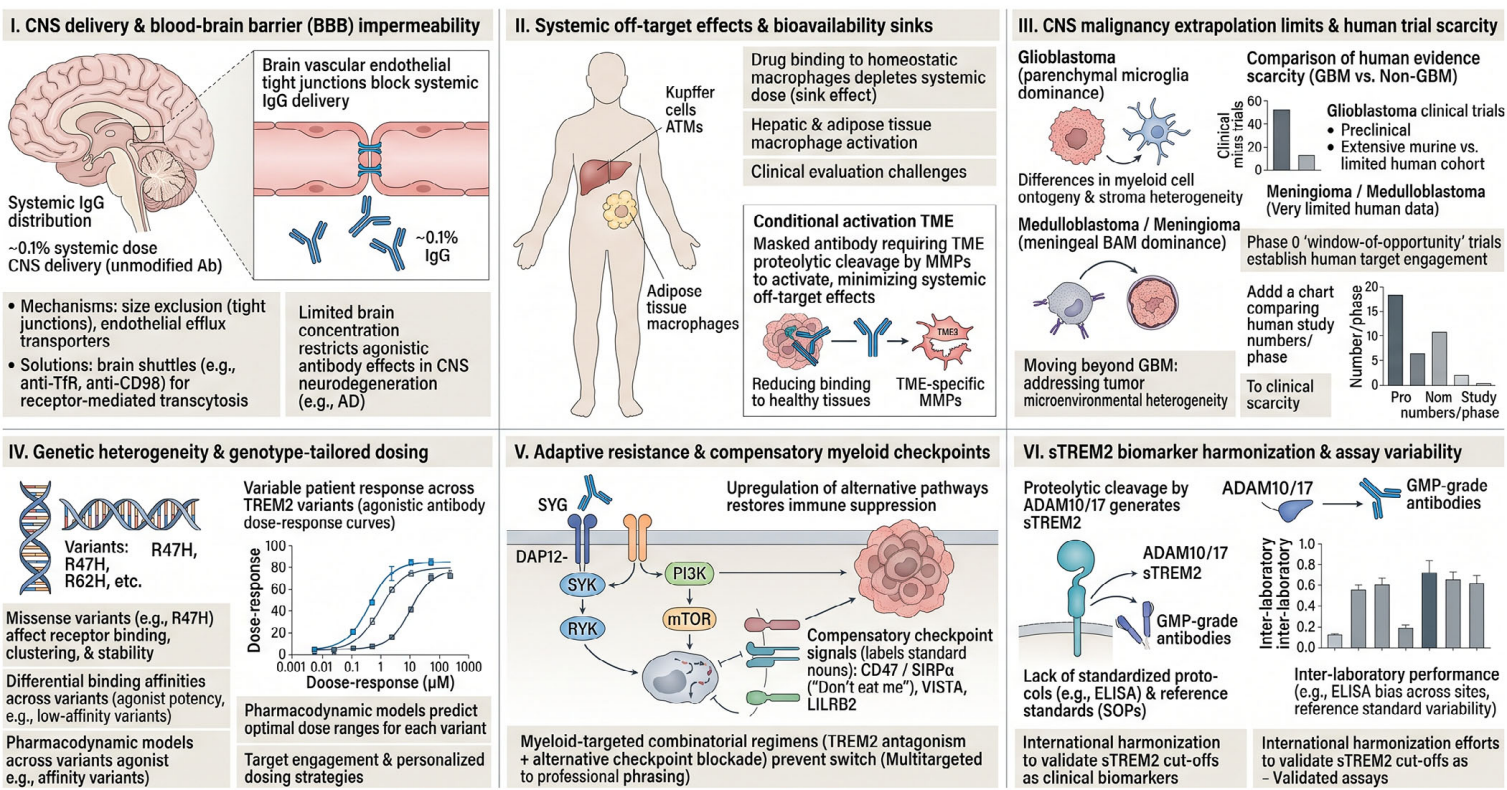
Six major translational barriers for TREM2 therapeutics. This comprehensive infographic delineates the six major translational hurdles facing TREM2‐targeted precision therapeutics, spanning physiological, pharmacodynamic and analytical challenges that impede clinical implementation. Physical obstacles remain paramount, notably the brain's unique physiology where tight vascular endothelial junctions create an impermeable BBB, severely restricting the CNS delivery of large molecule biologics like antibodies, which must be overcome through bioengineering innovations. Compounding delivery, systemic specificity is compromised as administered drugs are inadvertently bound to macrophages in healthy peripheral tissues, such as the liver and adipose tissue, generating ‘bioavailability sinks’ that reduce the therapeutic dose reaching the tumour bed and increasing the risk of off‐target toxicities. A newly expanded and critical barrier focuses on the conceptual limitation of relying disproportionately on preclinical murine GBM models, which do not easily extrapolate to other primary and secondary intracranial malignancies possessing drastically different immune microenvironments. This panel explicitly highlights the profound scarcity of human clinical trial evidence for TREM2‐targeted cancer therapies, presenting the extensive functional murine data versus the exceptionally limited human functionality models beyond GBM and mapping distinct developmental signatures that necessitate extreme caution when extrapolating results to paediatric tumours or brain metastases. Preclinical mechanistic assays are essential to determine whether TREM2 exerts tumour‐suppressive phagocytic effects or promotes tumour tolerance in these unselected non‐GBM contexts before initiating human therapeutic trials. Genetic variability adds another layer of complexity, as intrinsic coding variants, such as the R47H mutation that impairs ligand recognition, substantially alter receptor responsiveness, mandating a precision medicine‐oriented, genotype‐tailored approach to dose calibration. Clinical translation must also address the adaptive resilience of tumours, which circumvent initial myeloid checkpoint blockade by upregulating alternative, compensatory checkpoints and workaround signals, requiring rational, multi‐targeted combinatorial regimens to dismantle this shield. Finally, methodological and analytical challenges surround the clinical utility of sTREM2 as a primary biomarker, hindered by a severe lack of standardised assay protocols across laboratories. BBB, blood–brain barrier; CNS, central nervous system; GBM, glioblastoma; R47H, a TREM2 coding variant; sTREM2, soluble triggering receptor expressed on myeloid cells 2; TREM2, triggering receptor expressed on myeloid cells 2.

### 8.1 BBB penetration and CNS delivery

For CNS pathologies, including AD and GBM, the primary translational bottleneck is achieving sufficient target engagement within the brain parenchyma. Conventional large‐molecule biologics, such as IgG monoclonal antibodies, exhibit notoriously poor BBB penetrance, typically with less than .1% of the systemically administered dose reaching the CNS interstitial fluid.^145^ Consequently, achieving therapeutically active concentrations of TREM2 agonistic antibodies in the brain necessitates exceptionally high systemic doses, which are economically burdensome and biologically risky due to the potential for peripheral ‘sink effects’ by circulating monocytes and tissue macrophages.

To circumvent this, bioengineering strategies are rapidly evolving. The development of brain‐shuttle bispecific antibodies represents a major frontier. By fusing a TREM2‐activating moiety with a low‐affinity targeting arm directed against transcytosis receptors on the BBB—such as the Transferrin receptor (TfR) or CD98—researchers can facilitate active receptor‐mediated transport across the cerebral endothelium, exponentially increasing CNS bioavailability.^146^ Alternatively, AAV‐mediated gene delivery is being explored to induce localised, long‐term expression of TREM2 or its modulators directly within the CNS. However, AAV therapies are constrained by their own limitations, including vector immunogenicity, limited packaging capacity and the irreversible nature of the intervention, which complicates dosing termination in the event of adverse hyper‐inflammatory responses.^147^

### 8.2 Systemic specificity and off‐target immunosuppression

In the context of peripheral solid tumours, the therapeutic objective fundamentally inverts: the goal is to antagonise or deplete TREM2+ immunosuppressive TAMs to unleash cytotoxic T‐cell immunity. However, systemic administration of TREM2‐blocking or depleting antibodies carries significant risks of off‐target toxicity. TREM2 is not exclusively a tumour‐associated receptor; it plays a vital homeostatic role in various peripheral tissues, regulating lipid metabolism, debris clearance and the resolution of inflammation in specialised resident macrophages, such as hepatic Kupffer cells, ATMs and osteoclasts.^148^

Indiscriminate systemic blockade of TREM2 could inadvertently disrupt these homeostatic circuits, potentially precipitating severe systemic side effects, such as exacerbating atherosclerotic plaque instability, accelerating non‐alcoholic steatohepatitis (NASH) or impairing tissue repair following routine injury.^149^ To mitigate these off‐target effects, therapeutic optimisation must pivot towards conditional activation platforms. ‘Pro‐body’ therapeutics or conditionally active biologics are engineered with masking peptides that sterically hinder the antigen‐binding site. These masks are specifically cleaved and released only upon encountering the unique proteolytic landscape or the acidic, hypoxic milieu characteristic of the TME, ensuring that TREM2 blockade remains strictly localised to the tumour bed.

### 8.3 Extrapolation limits across CNS tumours and human trial scarcity

A critical conceptual limitation currently constraining the translational trajectory of TREM2‐directed oncology is the disproportionate reliance on preclinical models of GBM, coupled with an almost total absence of human clinical trial validation.^150^ While accumulating murine data suggest that microglial TREM2 sustains a tumour‐restraining, phagocytic state in GBM that synergises with ICB, treating this observation as a universal paradigm across neuro‐oncology risks substantial oversimplification.^107^ The fundamental barrier to extrapolating GBM‐derived findings to other CNS tumours lies in the profound ontogenetic, anatomical and immunological heterogeneity of the intracranial compartment.^151^ GBM represents an aggressively infiltrative parenchymal malignancy where the tumour‐associated myeloid stroma is predominantly dominated by CNS‐resident microglia and infiltrating bone marrow‐derived macrophages. In sharp contrast, non‐glioma intracranial tumours occupy drastically different microenvironmental niches that dictate alternative myeloid functional programs.

Specifically, meningiomas arise from the arachnoid cap cells of the leptomeninges and develop entirely outside the BBB within the dura mater and subarachnoid space. In this non‐parenchymal niche, the myeloid compartment is populated predominantly by dural border‐associated macrophages and circulating monocyte‐derived macrophages rather than yolk sac‐derived parenchymal microglia.^152^ Because peripheral monocyte‐derived macrophages typically co‐opt TREM2 to reinforce immunosuppressive and wound‐healing programs, TREM2 engagement in meningiomas might paradoxically promote stromal fibrosis and tumour tolerance rather than immune containment.^153^ Similarly, medulloblastomas, as the most prevalent paediatric malignant brain tumour arising in the posterior fossa, exhibit an immunologically cold architecture characterised by a low tumour mutational burden, minimal immune infiltration and distinct developmental signalling pathways.^154^ Whether TREM2‐mediated lipid clearance in the developing cerebellum exerts a tumour‐suppressive effect or inadvertently facilitates metabolic fuel scavenging for medulloblastoma stem cells remains entirely unknown due to the complete lack of dedicated syngeneic or genetically engineered in vivo models. Furthermore, secondary brain metastases originating from systemic primaries establish a highly destructive interface with the brain parenchyma where severe disruption of the blood–tumour barrier leads to a massive influx of peripheral monocytes that rapidly outnumber resident microglia, often reprogramming TREM2‐positive cells towards an aggressive pro‐angiogenic phenotype.^155^

The assumption that TREM2 agonism is universally therapeutic across all CNS malignancies also overlooks the fragile balance between microglial phagocytic clearance and secondary neurotoxicity. In primary brain malignancies, chronic or excessive activation of TREM2 signalling via agonistic biologics could trigger the indiscriminate engulfment of viable, stressed peritumoural neurons or exacerbate vasogenic cerebral oedema through the release of inflammatory matrix metalloproteinases and vascular permeability factors. In tumours with high lipid and myelin debris content, sustained TREM2‐driven lipid uptake might accelerate foam cell formation and exhaustion within TAMs, ultimately paralyzing anti‐tumour immunity.^156^ Without rigorous comparative validation across diverse primary and secondary intracranial tumour models, deploying TREM2‐activating therapies in unselected intracranial malignancies poses substantial clinical risks.

This mechanistic uncertainty is compounded by a profound scarcity of human clinical trial evidence evaluating TREM2‐targeted therapies in cancer. While the NDD pipeline has advanced to Phase 2 randomised clinical trials, such as the AL002 trial in early AD, with established CSF biomarker readouts, the oncology field remains in its earliest infancy.^140^ To date, only a handful of first‐in‐human Phase 1 trials evaluating antagonistic monoclonal antibodies, such as PY314, have been initiated for advanced peripheral solid tumours, while published clinical data regarding objective response rates, durable disease control and safety profiles remain extremely preliminary.^157^ More critically, there are currently zero active or completed clinical trials evaluating TREM2 agonists or antagonists in GBM or any other intracranial malignancy, meaning that every clinical assertion regarding TREM2 in human neuro‐oncology is entirely extrapolated from murine experimental models. Evaluating TREM2 engagement in human brain tumours also presents unique diagnostic hurdles, as standard neuro‐oncological imaging criteria frequently struggle to distinguish true tumour progression from immune‐mediated pseudo‐progression, while the surgical inaccessibility of serial brain biopsies prevents real‐time tracking of macrophage phenotypic shifts.^158^

To bridge this bench‐to‐bedside gap, the neuro‐oncology field must implement a structured and cautious translational roadmap. Preclinically, researchers must expand beyond standard GL261 glioma models to evaluate patient‐derived organotypic brain slice cultures and humanised mouse models of medulloblastoma, meningioma and brain metastases. Clinically, future drug development should prioritise Phase 0 window‐of‐opportunity surgical trials, in which administering a single sub‐therapeutic dose of a candidate TREM2 modulator prior to planned surgical resection allows direct mass spectrometry and ST evaluation of human resected tissue. Such clinical trial designs will formally establish whether target engagement, BBB penetration and myeloid reprogramming occur in the human brain before committing to large‐scale efficacy studies.

### 8.4 Genetic variability and genotype‐tailored dosing

Therapeutic responsiveness to TREM2 modulation is unlikely to be uniform across patient populations, heavily dictated by intrinsic genetic variability. Human genetic studies have identified numerous rare missense mutations in the TREM2 gene that fundamentally alter the receptor's extracellular structure, lipid–ligand‐binding affinity and downstream clustering efficiency. A therapeutic antibody designed to optimally cross‐link and activate wild‐type TREM2 might exhibit markedly reduced efficacy or entirely different binding kinetics in individuals carrying the R47H variant.^80^

This genetic heterogeneity mandates the integration of precision medicine principles into clinical trial design. Pharmacodynamic modelling must be customised, and patients must be genetically stratified prior to enrolment. As observed in early‐phase clinical trials evaluating TREM2 agonists (e.g., the AL002 trial for early AD), achieving optimal microglial activation requires precise dose calibration. Over‐stimulation in wild‐type carriers could inadvertently trigger a chronic, pro‐inflammatory neurotoxic state, whereas under‐dosing in mutation carriers may fail to rescue the phagocytic deficit.^159^ Thus, therapeutic optimisation relies on establishing genotype‐specific therapeutic windows.

### 8.5 Adaptive resistance in combination immunotherapy

While preclinical models demonstrate synergistic efficacy when combining TREM2 blockade with ICB like anti‐PD‐1 or anti‐CTLA‐4, clinical oncology has consistently shown that tumours possess remarkable plasticity and capacity for adaptive resistance. TAMs are highly dynamic; when their immunosuppressive function is constrained via TREM2 antagonism, the TME often responds by upregulating alternative compensatory myeloid checkpoints.^160^

Preclinical observations suggest that sustained TREM2 blockade may induce the compensatory activation of the CD47‐SIRPα ‘don't eat me’ axis, VISTA (V‐domain Ig suppressor of T‐cell activation) or CD47 (leukocyte immunoglobulin‐like receptor B2) pathways to re‐establish immune tolerance.^161^ Consequently, isolated TREM2 targeting is unlikely to provide durable remissions as a monotherapy. Future therapeutic strategies must proactively anticipate this resistance by designing rational, multi‐targeted combinatorial regimens. Formulating bispecific antibodies that simultaneously antagonise TREM2 and block compensatory checkpoints may be required to permanently dismantle the myeloid suppressive shield and maintain durable CD8+ T‐cell infiltration.^162^

### 8.6 Standardisation of sTREM2 as a clinical biomarker

A critical enabler of therapeutic optimisation is the development of robust, reliable biomarkers to monitor target engagement and disease progression.^163^ sTREM2, generated by ADAM10/17‐mediated proteolytic cleavage of the extracellular domain, has emerged as a leading candidate. In AD, CSF sTREM2 levels correlate with microglial activation, and early clinical trials have heavily relied on sTREM2 dynamics as a primary pharmacodynamic readout. Similarly, in oncology, elevated peripheral blood sTREM2 correlates with high intra‐tumoural macrophage burden and poor prognosis.^80^

Despite its immense promise, the clinical utility of sTREM2 is currently paralysed by severe methodological and analytical limitations. Currently, sTREM2 is predominantly quantified using electrochemiluminescence. While physiological CSF sTREM2 concentrations in healthy individuals typically range from 2000 to 6000 pg/mL and transiently elevate above 8000 pg/mL in prodromal AD cohorts, inter‐platform measurements often exhibit two to threefold discrepancies due to differing antibody epitopes and calibrators.^164^, ^165^ There is a profound lack of standardised detection protocols across different clinical laboratories. Variations in antibody clone specificity in enzyme‐linked immunosorbent assays (ELISA) or Single Molecule Array technologies, differences in pre‐analytical sample handling and the physiological variations between CSF and plasma sTREM2 pools lead to highly inconsistent datasets. To overcome this translational barrier, large‐scale, multi‐centre harmonisation consortia are urgently needed to establish standardised assay protocols, define universal reference materials and validate reproducible physiological and pathological cutoff values. Until such rigorous standardisation is achieved, the interpretation of sTREM2 as a predictive or prognostic biomarker in clinical trials remains fraught with ambiguity.^166^

## 9 CONCLUSION AND FUTURE PERSPECTIVES

Over the past two decades, TREM2 has evolved from an obscure myeloid surface receptor into an indispensable immunometabolic hub that coordinates innate immune sensing, lipid handling and functional plasticity across health and disease. As synthesised in this review, the functional output of TREM2 cannot be captured by rigid binary classifications—whether describing macrophage polarisation through outdated M1/M2 descriptors or conceptualising therapeutic modulation as a static ‘agonist versus antagonist’ dichotomy. Rather, TREM2 operates as a finely calibrated, context‐dependent rheostat whose biological trajectory is shaped by the intersection of four regulatory layers: cellular ontogeny, tissue and disease staging, local ligand and lipid architecture, and systemic microbial tuning along the gut–brain–tumour axis.

Unlocking the true translational potential of TREM2‐directed medicine demands a paradigm shift from broad, non‐selective receptor modulation towards biomarker‐guided, context‐tailored precision intervention. To bridge the formidable gap between preclinical discovery and clinical reality, the next generation of TREM2 translational research must prioritise four strategic frontiers:

1. Deconvoluting human microenvironmental heterogeneity: Given the stark scarcity of human clinical trial evidence outside of AD, therapeutic programs must avoid over‐relying on conventional murine models. Incorporating patient‐derived organotypic slice cultures, humanised platforms and Phase 0 ‘window‐of‐opportunity’ surgical trials will be essential to establish true target engagement and clarify whether TREM2 drives immune clearance or promotes tolerance within specific human lesions.
2. Next‐generation delivery and conditional biologics: Overcoming the physical barrier of the BBB for neurodegenerative indications requires the accelerated clinical development of receptor‐mediated transcytosis shuttles. Conversely, for peripheral oncology, minimising off‐target bioavailability sinks in healthy Kupffer cells and adipose tissue demands protease‐cleavable ‘pro‐body’ designs that restrict antagonistic antibody binding strictly to the acidic, protease‐rich TME.
3. Integrated multi‐omic companion stratification: The pronounced influence of intrinsic genetics and extrinsic variables mandates rigorous patient stratification. Metagenomic sequencing and functional immunophenotyping should be embedded into clinical protocols to identify responsive patient subsets and mitigate resistance.
4. International standardisation of sTREM2 bioassays: Before sTREM2 can fulfil its promise as a regulatory‐grade surrogate biomarker for target engagement or patient prognostication, the academic and diagnostic communities must establish harmonised reference calibrators and reconcile inter‐platform variability between electrochemiluminescent and digital single‐molecule technologies.

In summary, navigating the intricate nuances of TREM2 biology requires balancing mechanistic candour with therapeutic creativity. By decoding the precise spatial, temporal and metabolic signals that govern TREM2 signalling in individual tissue niches, rational immunotherapy can harness this master myeloid regulator to restore tissue homeostasis in neurodegeneration or dismantle immunosuppressive barriers in cancer, ultimately delivering meaningful clinical benefits to patients worldwide.

## AUTHOR CONTRIBUTIONS

Yaqing Lv: conceptualisation, formal analysis, writing—original draft. Mingpeng Shi: data curation, literature collection, figure preparation. Yongfei Zhao: investigation, reference collation, format arrangement. Yi Yu: methodology, critical reviewing draft, revision editing. Jiawei Lv: validation, resources, partial manuscript revision. Hai Zhao: conceptualisation, supervision, funding acquisition, writing—review and editing, project administration.

## CONFLICT OF INTEREST STATEMENT

The authors declare no conflicts of interest.

## ETHICS STATEMENT

Authors declare human ethics approval was not needed for this study.

## Data Availability

Not applicable.
